# Profiling cell dynamic changes of goat peripheral blood mononuclear cells after *Pasteurella multocida* infection with single-cell transcriptomics and histopathology

**DOI:** 10.1186/s13567-025-01661-2

**Published:** 2026-05-05

**Authors:** Qi An, Taoyu Chen, Junming Jiang, Hui Wu, Guansheng Wu, Zizhuo Jiao, Shiyuan Li, Yong Meng, Jiayang Tang, Yuanyuan Chen, Haoju Pan, Hong Li, Zhenxing Zhang, Yiwen Cheng, Yujing Fu, Zijing Huang, Riga Manchu, Qiaoling Chen, Fengyang Wang

**Affiliations:** 1 Hainan Key Laboratory of Tropical Animal Reproduction & Breeding and Epidemic Disease Research, Engineering Key Laboratory of Haikou, School of Tropical Agriculture and Forestry, Hainan University, 58 Renmin Avenue, Meilan District, Haikou, 570228 Hainan China; 2 School of Life and Health Sciences, Hainan University, Haikou, China

**Keywords:** Single-cell RNA sequencing, *Pasteurella multocida*, goat, PBMCs

## Abstract

Peripheral blood mononuclear cells (PBMCs) represent a critical component of the immune system, orchestrating both cellular and humoral responses while maintaining immunological homeostasis. This study investigates the PBMC immune response to *Pasteurella multocida* (*P. multocida*) infection in goats using single-cell RNA sequencing (scRNA-seq). PBMCs from goats before and after *P. multocida* infection were used for scRNA-seq), and pathological examinations were performed on different tissues of the infected hosts. Results demonstrated that recombinase polymerase amplification—lateral flow dipstick (RPA-LFD) detected *P. multocida* in peripheral blood and lungs, with histopathology confirming lesions. A total of 42 615 cells were annotated as nine cell subgroups, revealed dynamic alterations in immune cell populations, including significant increase in B cells and monocytes alongside decreased T cells and megakaryocytes post-infection. Further analysis identified 48 differentially expressed genes consistently modulated across comparison groups, with significant enrichment in immune-related Gene Ontology (GO) terms, as well as Kyoto Encyclopedia of Genes and Genomes (KEGG) pathways including IL-17 signaling pathway and cytokine–cytokine receptor interactions. Pseudotime trajectory uncovered four B cell and two T cell differentiation lineages. Inter-cell communication exhibited enhanced activity post-infection. Integration with RNA-seq data revealed key genes (*IL1R2*, *RBM34*, *EDEM3*, and *MZB1*) potentially critical in early infection. These findings substantially advance the understanding of PBMCs-mediated immune responses during the early stage of *P. multocida* infection in goats, identifying key genes and immune pathways. The study not only provides valuable insights into goat immunology but also establishes a foundation for developing improved diagnostic tools and targeted therapeutic interventions against this economically significant pathogen.

**Supplementary Information:**

The online version contains supplementary material available at 10.1186/s13567-025-01661-2.

## Introduction

As a zoonotic opportunistic pathogen, *Pasteurella multocida* (*P. multocida*) can infect humans, domestic and wild animals [1]. This bacterium commonly colonizes the oral, nasal cavities and upper respiratory tract of animals, Human infections typically occur through animal bites, scratches, or contact with animal nasal secretions [2, 3]. After exposure to the pathogen, it may cause severe pneumonia, sepsis and even death in patients with weak resistance [3, 4]. Pediatric infections are particularly concerning, as children may develop bacterial meningitis, conjunctivitis, and osteoarthritis [5]. Beyond its impact on human health, Pasteurellosis poses a major threat to livestock industries, where it causes economically devastating outbreaks. In animals, the pathogen primarily induces respiratory infections [6], while in goats and sheep, it can also cause hemorrhagic septicemia—a highly fatal condition [7].

Goats are one of the earliest domesticated animals, and remain critically important for agriculture worldwide. The Hainan black goat, a native breed indigenous to Hainan Province, is particularly valued for its high-quality delicious meat, robust resistance and environmental adaptability [8]. These desirable traits have led to a large market demand in China. However, due to the high temperature and humidity in Hainan island, Hainan black goats are susceptible to respiratory diseases caused by *P. multocida.* Therefore, understanding the pathogenic mechanisms of *P. multocida* in Hainan black goats represents a critical research priority with important implications for animal health management and sustainable livestock production.

Single-cell RNA sequencing (scRNA-seq) has emerged as a transformative technology for characterizing transcriptomes at single-cell resolution. In goats and sheep research, current applications of scRNA-seq have mainly focused on hair follicles [9, 10], germ cells [11–17], and development [18–21]. Notably, Chen et al. employed scRNA-seq to identify putative severe acute respiratory syndrome coronavirus 2 (SARS-CoV-2) target cells in goat lung tissue by characterizing cells co-expressing ACE2 and TMPRSS2, although without direct viral challenge [22]. In addition, Wang et al. applied this technology to investigate the immune response of goat peripheral blood mononuclear cells (PBMCs) to *Haemonchus contortus* [23]. Despite these advances, the application of scRNA-seq to study bacterial pathogenesis in goats remains strikingly limited.

PBMCs are composed of circulating multifunctional immune cell types, which play important roles in mediating cellular and humoral immune responses, maintaining immune homeostasis, and reflecting the real-time immune status of the body [24]. In addition, PBMCs have multi-differentiation potential because they contain a variety of multipotent progenitor cell populations [25]. This is beneficial to tissue regeneration and functional repair after injury. *P. multocida* causes hemorrhagic septicemia in goats, and microorganism invasion in the bloodstream is characteristic of this disease. PBMCs activation is thought to play an important role in orchestrating this immune response owing to their positive changes to the transcriptome and secretory profile [26]. Thus, in this study, conducting scRNA-seq on PBMCs of goats infected with *P. multocida* is conducive to our in-depth understanding of the immune mechanism of the host against this bacterium.

## Materials and methods

### Identification of *P. multocida* HN01 strain and preparation of the infection solution

The serotype D *P. multocida* strain HN01 (GenBank Accession Number: CP037861.1) used in this study was previously isolated from the lung tissue of a Hainan black goat that had died of pneumonia [27]. The strain is stored at the Hainan Key Lab of Tropical Animal Reproduction, Breeding and Epidemic Disease Research. For the identification of the *P. multocida* HN01 strain, in brief, after resuscitation, a monoclonal colony was selected for *KMT1* gene (*P. multocida* conserved gene) and *dcbF* gene (serotype D specific gene) amplification. The correctly identified colony was then inoculated into Tryptic Soy Broth (TSB) (Hopebio, China), supplemented with 5% (v/v) bovine serum (Sijiqing, China) and incubated overnight at 37 ℃ before Gram staining. For growth characterization, 200 μL of bacterial suspension (OD_600nm_≈0.6) was inoculated into 20 mL TSB containing 5% bovine serum, and OD_600nm_ values were recorded at 2 h intervals over 24 h. Concurrently, bacterial cultures collected at 4 h, 6 h, 8 h, and 10 h were diluted by gradient, and 100 μL suspension of the 10^5^, 10^6^,10^7^ dilutions was coated on soybean-casein digest agar medium (TSA) supplemented with 5% (v/v) bovine serum. In total, three replicate plates were prepared for each dilution. The colonies were counted after overnight culture at 37 ℃. The growth curve and linear equation of the *P. multocida* HN01 strain were plotted according to OD_600nm_ values at 24 h and colony counting results. On the basis of the above results, the infection solution containing 2 × 10^9^ CFU of *P. multocida* HN01 in 250 μL of sterilized phosphate-buffered saline (PBS) was prepared.

### Clinical examination of goats

Five clinically healthy, 4-month-old male Hainan black goats weighing 18 ± 2 kg were initially selected from a local goat farm (ChuXin Farm, Dingan, Hainan Province, China). Following a 2- week health monitoring period, two goats were enrolled in the study. In brief, the temperatures of the inner thigh and anus were measured with a veterinary thermometer, and the cumulative clinical score (CCS) of lung was assessed according to Kacar et al. [28]. Nasal swabs and jugular blood samples were collected every 7 days for 2 weeks before purchasing the goats. Each nasal swab was inserted into 0.5 mL ddH_2_O and stirred. The suspension was placed in a metal bath at 100 ℃ for 5 min and subsequently used for PCR detection of pathogenic bacteria. Primers and cycling conditions for PCR are listed in Additional files 1 and 2. Jugular blood samples were divided for four distinct analyses: one for routine blood examination (Yuanji Pet Hospital, Haikou, Hainan Province, China), one for DNA extraction, and two for serological testing or Rose Bengal Plane Test (RBPT) and ELISA, respectively, according to the manufacturer’s procedures.

### Animal management and sampling

The experimental goats were housed in separate sterile pens with age-appropriate temperature and humidity levels during the test period. They were fed a typical standard control diet with free access to food and water.

Jugular blood from each goat was collected as a control group (CK group) before the injection of *P. multocida*. Following intratracheal injection of 250 μL *P. multocida* HN01 bacterial suspension, serial blood collections were performed at 4 h intervals for blood routine examination, with concurrent monitoring of body temperature and CCS. The 24 h post-injection samples were designated as the experimental group (Pm group). Goats were then euthanized for tissue collection, including heart, liver, spleen, lung, and kidney. Each tissue was divided into three parts. One was frozen at −80 ℃ for long-term storage, one was used to extract DNA for recombinase polymerase amplification—lateral flow dipstick (RPA-LFD) detection, and the other placed in universal tissue fixative (G1101, Servicebio, China) for histopathological examination following hematoxylin and eosin (HE) staining. An additional file shows the details of the RPA-LFD assay for toxigenic *P. multocida* (see Additional file 3).

### ScRNA-seq and data processing

PBMCs were isolated from jugular blood samples of both the CK and Pm groups using a density gradient centrifugation method with a peripheral blood lymphocyte isolation kit (Solarbio, Beijing, China). Cell viability was assessed by mixing 10 μL of single-cell suspension with an equal volume of 0.4% trypan blue solution, followed by manual counting and adjustment to achieve optimal live cell concentration. Cell suspensions were loaded on a 10 × Genomics GemCode Single-cell instrument that generates single-cell Gel Bead-In-EMlusion (GEMs). Upon dissolution of the Gel Bead within each GEM, sequencing adapters were released and mixed with cell lysate and Master Mix. Barcoded, full-length cDNAs were then reverse-transcribed from poly-adenylated mRNA. Libraries were generated from the cDNAs using the Chromium Next GEM Single Cell 3′ Reagent Kits v3.1 and high-throughput sequencing was performed subsequently by using Illumina sequencing platform.

10 × Genomics Cell Ranger software (version 3.1.0) was used to convert raw BCL files to FASTQ files, alignment and counts quantification. The cell by gene matrices for each sample were individually imported into Seurat [29] version 3.1.1 for downstream analysis. Low-quality cells were included as those exhibiting an abnormally high number of UMIs (≥ 21 000), a mitochondrial gene percentage ≥ 20%, and either fewer than 290 or more than 4400 detected genes. After removing these low-quality cells from the dataset, Harmony was used for data consolidation, batch effect correction and cell clustering [30]. Uniform Manifold Approximation and Projection (UMAP) was then used to visualize the resulting clusters [31].

### Cell type annotation

Cell type annotation was performed through the comprehensive integration of PanglaoDB [32] and CellMarker [33] databases, complemented by manual curation of published literature. B cells and T cells were re-clustered by the R language Seurat package, and the re-clustered cells were annotated based on their highly expressed specific genes.

### Potential marker genes analysis

To analyze the potential marker genes of each type of PBMCs, the up-regulated genes of each cell type in CK_1 (uninfected goat No. 5), CK_2 (uninfected goat No. 3), Pm_1 (*P. multocida* infected goat No. 5) and Pm_2 (*P. multocida* infected goat No. 3) samples were screened by |Log_2_(Fold Change)|≥ 1 and FDR < 0.01, respectively. The up-regulated genes identified in each cell type were individually compared with those of every other cell type. Genes that were also found to be up-regulated in any other cell type were excluded. The final retained set of genes was exclusively up-regulated in that specific cell type. These genes of the four groups were intersected, and these genes were used as potential marker genes of PBMCs cells.

### Gene Ontology (GO), Kyoto Encyclopedia of Genes and Genomes (KEGG) and enrichment analysis of differentially expressed genes (DEGs)

DEGs analysis was performed between comparison groups CK_1 versus Pm_1 and CK_2 versus Pm_2 using stringent thresholds of |Log_2_(Fold Change)|≥ 1 and FDR < 0.05. The analysis identified distinct sets of DEGs for each paired comparison. Additionally, we extracted the common DEGs shared between both comparison groups. Subsequently, GO and KEGG enrichment analyses were systematically conducted on these three distinct gene sets: the CK_1 versus Pm_1, CK_2 versus Pm_2, and their intersection.

### Gene set score analysis

On the basis of the KEGG enrichment results, we focused on inflammation- and immunity-related pathways. The relevant gene sets were systematically retrieved from three pathway databases: KEGG [34], PathCards [35], and Reactome [36]. We then compared these genes with the human and mouse genomes to convert them into the corresponding gene names of goats. The AddModuleScore function of the Seurat package was used to calculate gene set scores for selected cell subsets. 

### Pseudotime trajectory analysis

Pseudotime trajectory analysis was conducted to investigate the developmental dynamics of B- and T- cell subsets. On the basis of the single-cell gene expression matrix and the expression of cyclic characteristic protein genes in cells, the AddModuleScore function of Seurat software was used to score the possible cycle periods of B and T cell subsets. Initial cell states for trajectory reconstruction were determined through integration of these cell cycle predictions with established biological knowledge from published literature [37–39]. Developmental trajectories of B and T cells were reconstructed using R language Slingshot package [40]. Gene expression patterns along these trajectories were analyzed with TradeSeq [41], identifying significantly DEGs (|Log_2_(Fold Change)|≥ 1 and *P*-vaule < 0.01) across distinct differentiation lineages. These genes were then analyzed for GO and KEGG enrichment.

### Ligand-receptor intercellular communication network analysis

Intercellular communication analysis was performed using CellChat [42] to systematically compare ligand–receptor interaction networks between CK and Pm groups. The computational analysis mainly comprised the following four steps: (1) Combine gene expression with the interactions between signaling ligands, receptors, and their cofactors contained in the software to simulate the probability of intercellular communication. (2) Comparison of all cell types in the dataset and analysis of the number of significantly enriched ligand–receptor interactions between the two cell types to determine their biological relevance. (3) On the basis of the above analysis of the expression of ligand–receptor interactions, the sum of the number of ligand–receptor interactions and the probability of communication between two cells was obtained, and the communication relationship between cells was initially evaluated. (4) The interaction was considered significant if* P*-value < 0.05. The probability of intercellular ligand–receptor communication was calculated according to the communication probability of the calculated signaling pathway.

### RNA-seq of goat peripheral blood

Three goats (No. 2, 3, and 5) that had passed the prior health screening were experimentally infected with *P. multocida* according to the infection method and dosage described previously. Peripheral blood samples were collected via jugular vein at pre- and post-infection time points. Total RNA was extracted from these samples using TRNzol Universal Reagent (Tiangen, Beijing, China) following the manufacturer’s protocol. RNA-seq was performed by Panomic Biopharmaceutical Technology Co., Ltd (Shanghai, China) on an Illumina platform. Raw sequencing data underwent quality control processing to generate high-quality Clean Data. The filtered Reads were then mapped to the reference genome using HISAT2 software to analyze the gene expression level. DEGs analysis was conducted with thresholds of |Log_2_(Fold Change)|> 1 and *P*-value < 0.05. The resulting DEGs were subjected to functional annotation through GO and KEGG. For transcription factor prediction, DEGs were cross-referenced with the AnimalTFDB [43] database to identify putative transcription factors and classify them into respective families. The number of differentially expressed transcription factors within each family was subsequently quantified. Randomly selected DEGs were validated by qRT-PCR. In brief, reverse transcription of RNA was performed using FastKing gDNA Dispelling RT SuperMix (Tiangen, Beijing, China), followed by qPCR with SYBR Green Pro Taq HS Premix PCR reagent (Accurate Biology, Hunan, China). Gene expression levels were calculated using the 2^−ΔΔCT^ method. Details regarding primer sequences, reaction mixtures, and thermal cycling conditions are provided in Additional files 4 and 5.

### Integrated analysis of scRNA-seq and RNA-seq

DEGs were systematically re-screened from both PBMCs scRNA-seq data and pulmonary transcriptomic profile at 24 h post-infection [44], using consistent statistical thresholds (|Log_2_(Fold Change)|> 1 and *P*-value < 0.05). These redefined DEG sets were subsequently integrated with the previously generated peripheral blood RNA-seq dataset for comparative analysis.

## Results

### Clinical examination results of goats

**Figure 1:**
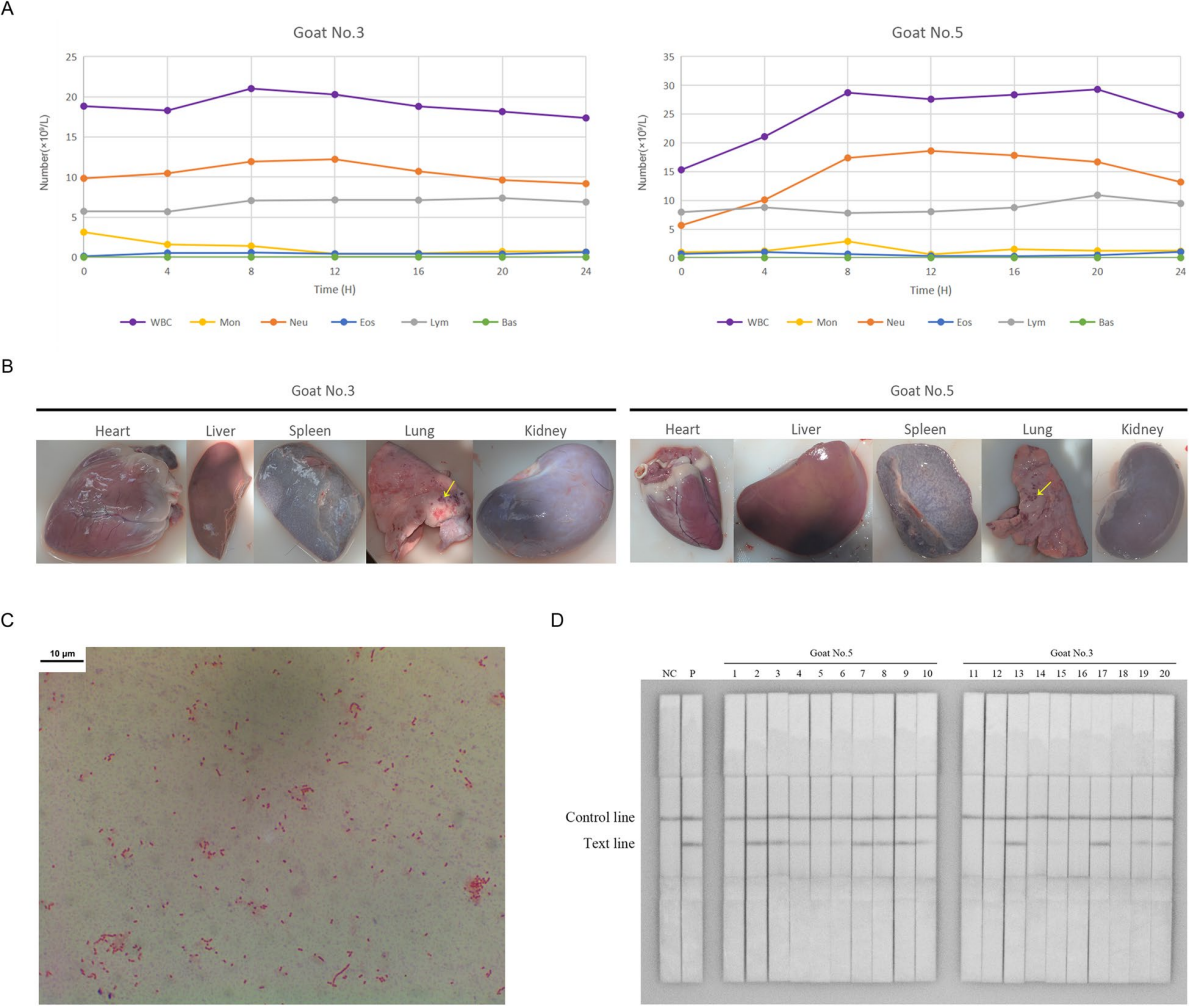
**Post-infection monitoring of goats challenged with *P. multocida***. **A** Routine blood examination of goats during 24 h post-infection period. **B** Organ lesions in goats infected with ***P. multocida***. **C** Gram staining of bacterial isolates from the lung tissues of infected goats. **D** RPA-LFD detection of peripheral blood pre- and post-infection, and post-infection organ tissues of challenged goats. WBC: white blood cell; Mon: monocyte; Neu: neutrophil; Eos: Eosinophil; Lym: lymphocyte; Bas: basophil; NC: negative control; P: positive control; 1 and 11: pre-infected peripheral blood; 2 and 12: post-infection peripheral blood; 3 and 13: heart; 4 and 14: liver; 5 and 15: spleen, 6 and 16: kidney, 7 and 17: left lung (non-lesion); 8 and 18: left lung (lesion); 9 and 19: right lung (non-lesion); 10 and 20: right lung (lesion).

During the 2-week health monitoring period, all five goats remained healthy, with nasal swab detection confirming the absence of six specific pathogenic bacterial infections (see Additional files 6A–F and 26). The optimized RPA-LFD detection method (see Additional files 3 and 26) and ELISA yielded negative results for *P. multocida* infection (see Additional files 6G and 7)*.* Similarly, RBPT (see Additional file 6H) and ELISA analyses (Additional file 7) demonstrated no evidence of *Brucella* infection. Additional testing for *Mycoplasma* and *Chlamydia* also produced negative results (Additional file 7). On the basis of comprehensive evaluation of CCS for lung examination and routine blood tests (Additional file 8), two goats (No. 3 and No. 5) were selected for subsequent *P. multocida* challenge experiment. According to the growth curve and linear equation, the two selected goats were infected with confirmed *P. multocida* (see Additional files 9 and 26). Clinical monitoring over 24 h using CCS revealed the onset of coughing and respiratory distress within four hours post-infection. At 20 h post-infection, the body temperature of infected goats began to rise. Goat No. 3 exhibited increased ocular secretions after eight hours of infection. The routine blood tests (Figure 1A) showed lymphocyte counts peaked at 20 h post-infection then declined, while neutrophils reached maximum levels at 12 h. Necropsy examination demonstrated significant pulmonary pathology characterized by multiple hemorrhagic foci (Figure 1B). *P. multocida* was detected in lung tissues by spread plate culture and further confirmed by Gram staining (Figure 1C), and *16S rRNA* sequencing, which showed more than 99% sequence homology with *P. multocida* HN01 strain. The RPA-LFD assay demonstrated the presence of *P. multocida* in both peripheral blood and cardiac tissues of the two infected goats (Figure 1D). Hepatic and renal tissues from goat No. 5 also tested positive for the pathogen. Notably, the lesioned left lung tissue from goat No. 3 was the only pulmonary sample that tested negative, as all the other lung tissue samples from the two goats were confirmed to harbor *P. multocida.*

### HE stain results

**Figure 2:**
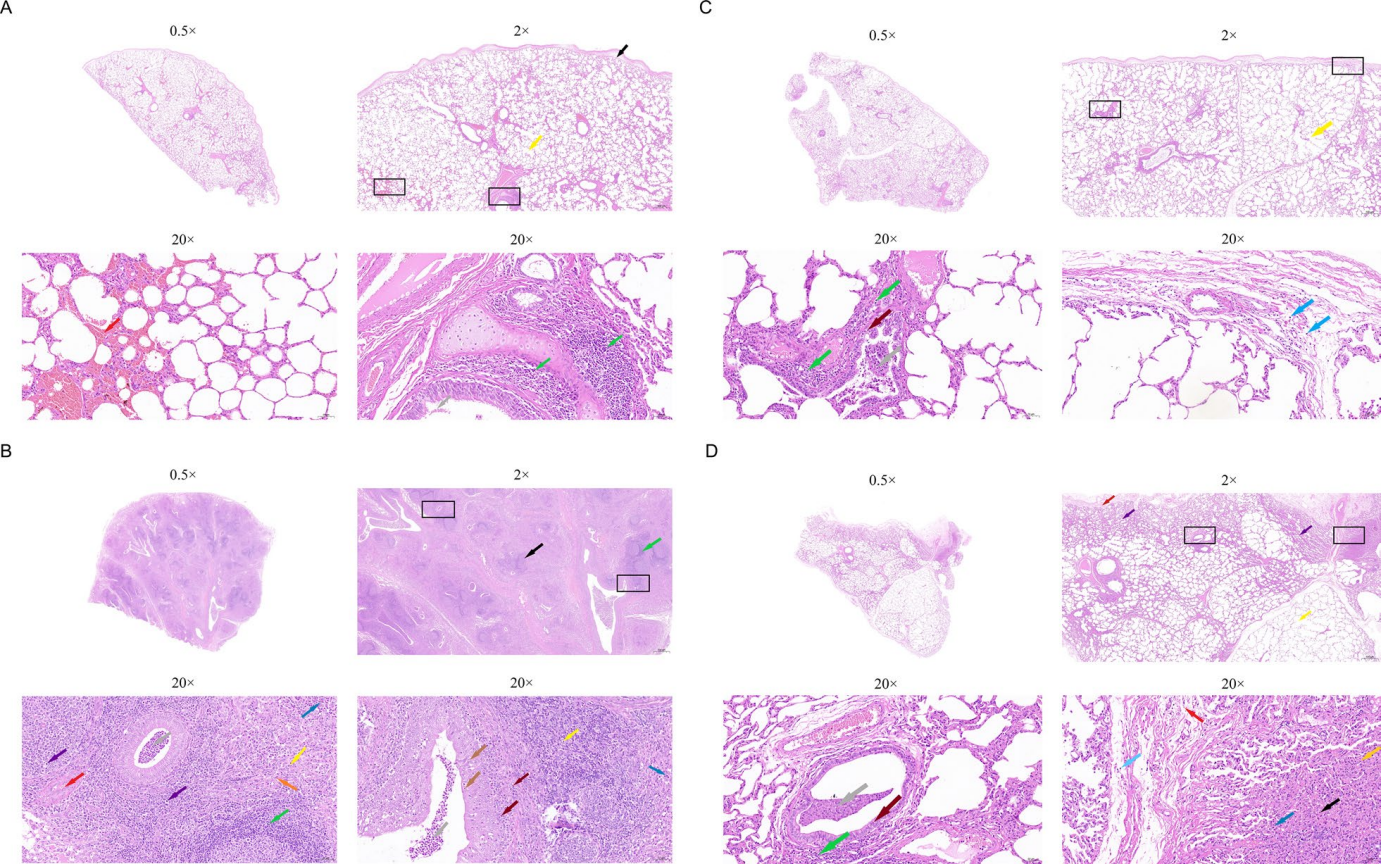
**HE staining of right lung tissues from two goats following** ***P. multocida*** **infection**. **A** and **C** The HE staining of non-lesioned right lung tissues from goat No. 3 (**A**) and goat No. 5 (**C**). **B** and **D** The HE staining of lesioned right lung tissues from goat No. 3 (**C**) and goat No. 5 (**D**).

Histopathological examination of the hearts, livers, spleens, kidneys, and lungs from the affected goats revealed varying degrees of tissue damage across multiple organs (Figure 2, Additional files 10 and 11).

The heart tissue of goat No. 3 showed widened intermuscular connective tissue spaces between myocardial fibers (black arrows), though the hearts overall exhibited minimal pathological changes (see Additional file 10A). In the livers, hepatocyte edema (black arrows) and vascular congestion (yellow arrows) were observed (see Additional file 10B). The spleens showed notable differences between the two goats. Goat No. 5 presented extensive white pulp hyperplasia with occasional adhesions (black arrow). In contrast, goat No. 3 had reduced white pulp but prominent granulocyte infiltration (blue arrows) (see Additional file 10C). Renal pathology also differed between the goats. Goat No. 3 demonstrated renal capsular dilatation (blue arrow), hydropic degeneration of renal tubular epithelial cells (black arrow), interstitial hemorrhage (orange arrow), and eosinophilic material within tubular lumens (yellow arrow). Goat No. 5 showed milder changes, including glomerular and renal tubular dilatation (blue and red arrows, respectively), intraluminal eosinophilic material (yellow arrows), and perivascular edema (orange arrow) (see Additional file 10D). Notably, no significant inflammatory cell infiltration was detected in the hearts, livers, spleens, or kidneys.

Histopathological analysis of grossly observed non-lesioned and lesioned regions from both right and left lungs of goats was performed following HE staining. In goat No. 3 (Figure 2A), analysis of the non-lesioned right lung section demonstrated loosely arranged subcapsular connective tissue (black arrow) with multiple dilated alveoli (yellow arrow). Focal mild alveolar hemorrhage (red arrow) was observed, with small numbers of erythrocytes visible within alveolar spaces. The bronchial lumen contained minimal exfoliated epithelial cells (gray arrow), while occasional small peribronchiolar lymphocyte and granulocyte infiltrates (green arrow) were noted. Microscopic examination of the non-lesioned left lung tissue (see Additional file 11A) revealed additional pathological alterations including moderate extent alveolar narrowing (purple arrows), irregular arrangement of bronchiolar epithelial cells (brown arrows), and vascular stasis (orange arrows). The lesioned areas displayed more severe pathological changes compared with grossly normal regions. In the right lung lesioned tissue (Figure 2B), widespread pulmonary parenchymalization (black arrow) was observed along with proliferation of fibrous connective tissue (orange arrow). Necrotic cellular debris (yellow arrow) could be seen with visible nuclear division (purple arrow) and pyknotic or fragmented nuclei (brown arrow). Extensive infiltration of granulocytes and macrophages (blue arrow) was present. Bronchial lumina contained numerous granulocytes and cellular debris (gray arrow), while small numbers of lymphocyte infiltration were observed in both the bronchial mucosal layer and lamina propria (dark red arrow). Numerous blood vessels exhibited luminal narrowing with endothelial cell hyperplasia (red arrow), accompanied by focal lymphocyte infiltration surrounding bronchioles and blood vessels (green arrow). In contrast, the left lung lesioned tissue (see Additional file 11B) contained fewer necrotic epithelial cells but showed more lymphocyte and granulocyte infiltration in the bronchial mucosa and lamina propria (dark red arrow). The histopathological characteristics in lung tissues from goat No. 5 (Figure 2C and D, Additional file 11C and D) were similar to those in goat No. 3.

### Data quality of scRNA-seq

**Figure 3:**
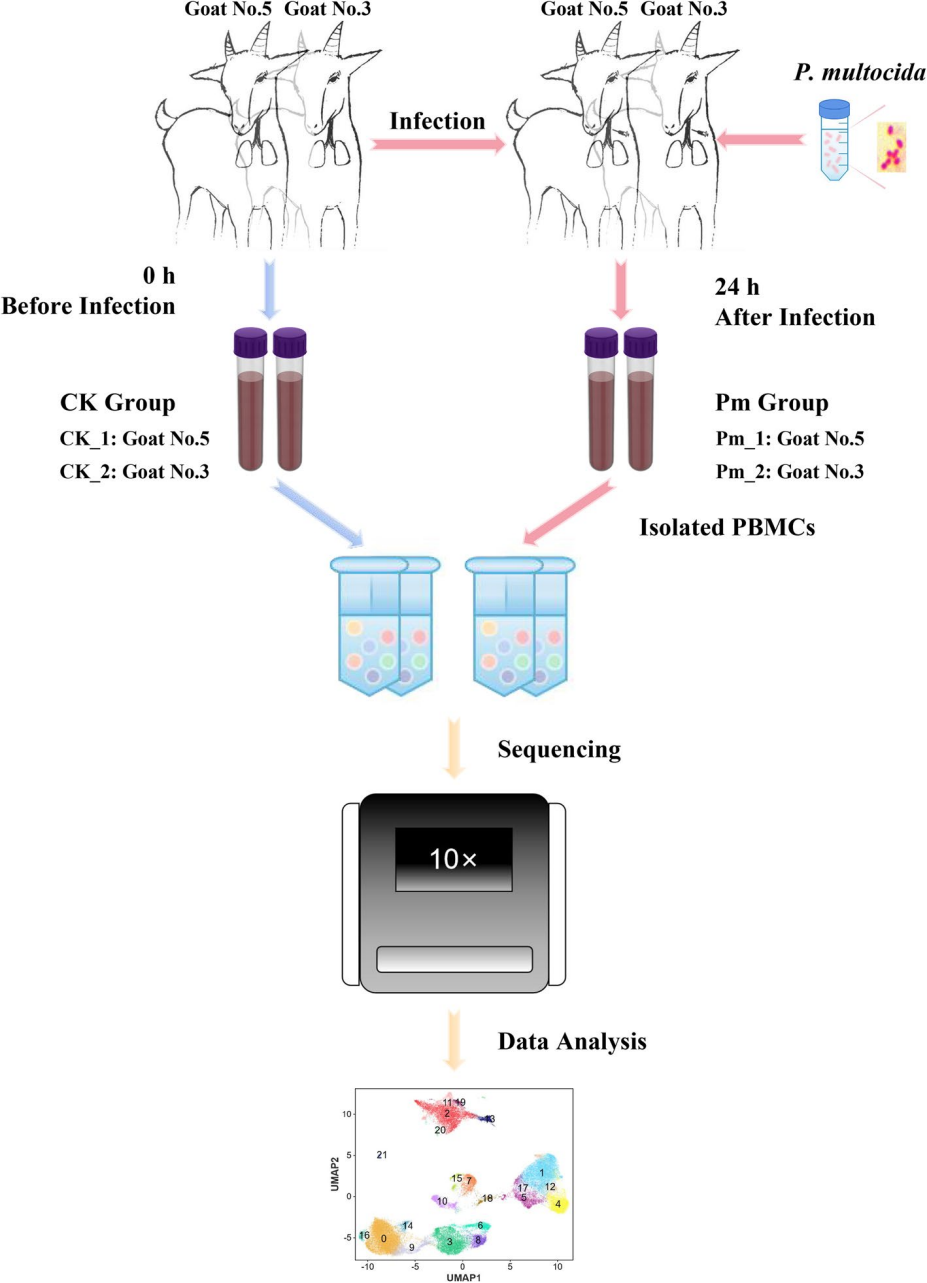
**Experimental workflow for scRNA-seq analysis of PBMCs from** ***P. multocida*-infected goats**. Goats were challenged with ***P. multocida*** via intratracheal injection. Jugular venous blood was drawn at pre-infection (0 h) and post-infection (24 h) timepoints. PBMCs were purified by density gradient centrifugation. Viable PBMCs were processed for scRNA-seq using 10 × Genomics platform.

As shown in Figure 3, the jugular venous blood of Hainan black goats was collected before or after the infection. The isolated PBMCs demonstrated > 90% viability through trypan blue staining. Following adjustment to appropriate concentration, the cell suspensions were processed for scRNA-seq. Sequencing data analysis was performed on 11 639, 11 205, 12 965, and 11 665 cells in the CK_1, CK_2, Pm_1, and Pm_2 groups (33 778, 35 981, 37 665, and 38 036 reads per cell, respectively) based on the Cell Ranger screening results (Table 1). The alignment rates to the reference genome exceeded 89% across all samples. All samples exhibited superior data quality with Q30 scores above 92% for Barcode, RNA reads and UMI. After further filtration, 10 430, 10 119, 11 504, and 10 562 cells in the CK_1, CK_2, Pm_1, and Pm_2 groups were used for downstream analysis.

**Table 1:** **ScRNA-seq data quality statistics of the CK and Pm groups**

Projects	CK_1	CK_2	Pm_1	Pm_2
Number of reads	393137004	403164654	488333163	443693483
Valid barcodes	96.0%	96.5%	96.6%	96.9%
Sequencing saturation	70.9%	63.5%	68.8%	66.1%
Q30 bases in barcode	97.1%	96.9%	96.8%	97.0%
Q30 bases in RNA read	93.0%	92.6%	93.2%	93.0%
Q30 bases in UMI	96.7%	95.9%	96.4%	96.6%
Number of cells (preliminary filtration)	11639	11205	12965	11665
Fraction reads in cells (preliminary filtration)	90.1%	93.0%	90.9%	91.0%
Mean reads per cell (preliminary filtration)	33778	35981	37665	38036
Median genes per cell (preliminary filtration)	1419	1663	1542	1601
Total genes detected (preliminary filtration)	17908	18073	18102	17855
Median UMI counts per cell (preliminary filtration)	3050	3906	3733	3926
Reads mapped confidently to genome (preliminary filtration)	89.0%	89.6%	89.5%	89.6%
Reads mapped confidently to intergenic regions (preliminary filtration)	27.8%	28.9%	28.0%	29.6%
Reads mapped confidently to intronic regions (preliminary filtration)	31.5%	28.8%	26.9%	24.1%
Reads mapped confidently to exonic regions (preliminary filtration)	29.7%	32.0%	34.5%	35.9%
Reads mapped confidently to transcriptome (preliminary filtration)	49.0%	49.9%	50.7%	51.5%
Number of cells (further filtration)	10430	10119	11504	10562
Median genes per cell (further filtration)	1372	1575	1470	1514
Median UMI counts per cell (further filtration)	2896.5	3605	3477	3593.5

### Goat peripheral blood cell type identification and clustering analysis

**Figure 4:**
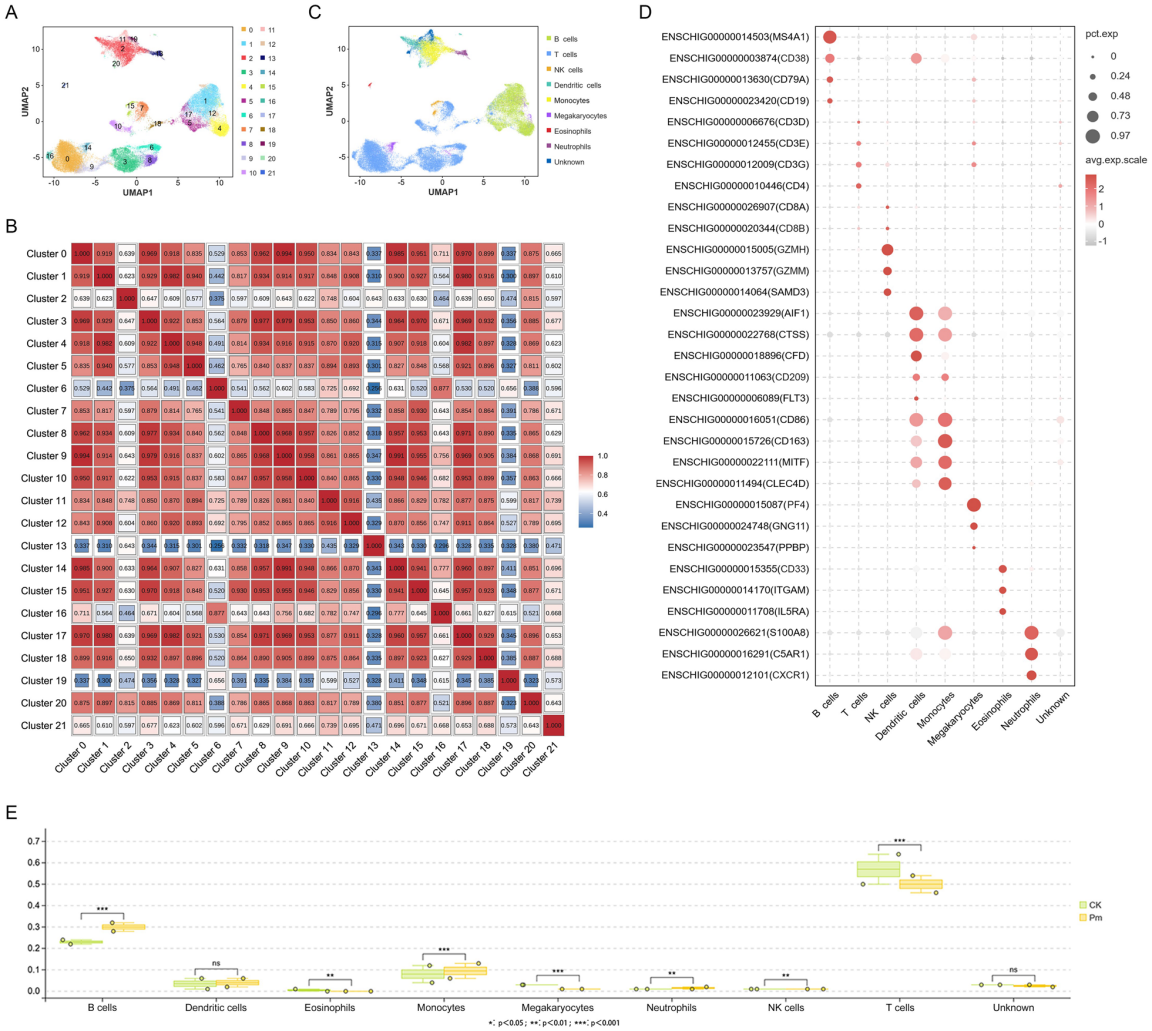
**Single-cell atlas of PBMCs of goats infected with** ***P. multocida*** **HN01**. **A** UMAP dimensionality reduction obtained 22 clusters of cells identified in goat PBMC pool. **B** In the correlation heatmap, a redder color represents a stronger correlation between two clusters, and a bluer color represents a weaker correlation between two clusters. **C** UMAP displaying all annotated nine cell types. **D** Cell type annotation and bubble plot representing marker genes (y-axis) in each cluster (x-axis). Bubble size represents the proportion of genes expressed in the cluster; color represents the normalized value of the average gene expression in the cluster. **E** Chi-square test of nine cell types before and after infection with *P. multocida.*

To determine the cell types in goat PBMCs, Harmony and PCA were performed. As shown in Figure 4A, 42 615 single cells were segregated into 22 distinct clusters through unsupervised clustering. On the basis of the marker genes of cell types in PBMCs reported in the literature, the CellMarker and PanglaoDB databases, and supplemented by inter-cluster correlation analysis (Figure 4B), the 22 clusters were subsequently annotated as nine cell populations (Figures 4C and D), with one population remaining unknown due to the absence of uniquely expressed marker genes. To comparatively evaluate the impact of *P. multocida* infection across distinct cell populations within goat PBMCs, the cell numbers for each cell type were counted in both CK and Pm groups (Table 2). Chi-square testing (Figure 4E) indicated a statistically significant increase in B cell and monocyte populations following bacterial challenge, concomitant with marked reductions in T cell and megakaryocyte counts.

**Table 2:** **Frequency statistics of each cell type**

Cell type	CK_number	CK_pct (%)	Pm_number	Pm_pct (%)	Trend
B cells	5647	27.48	7629	34.57	Up
T cells	10854	52.82	10120	45.86	Down
NK cells	254	1.24	202	0.92	Down
Dendritic cell	723	3.52	853	3.87	Up
Monocytes	1592	7.75	2083	9.44	Up
Megakaryocytes	572	2.78	220	1	Down
Eosinophils	108	0.53	72	0.33	Down
Neutrophils	236	1.15	318	1.44	Up
Unknown	563	2.74	569	2.58	Down
Total	20549	100	22066	100	

**Figure 5:**
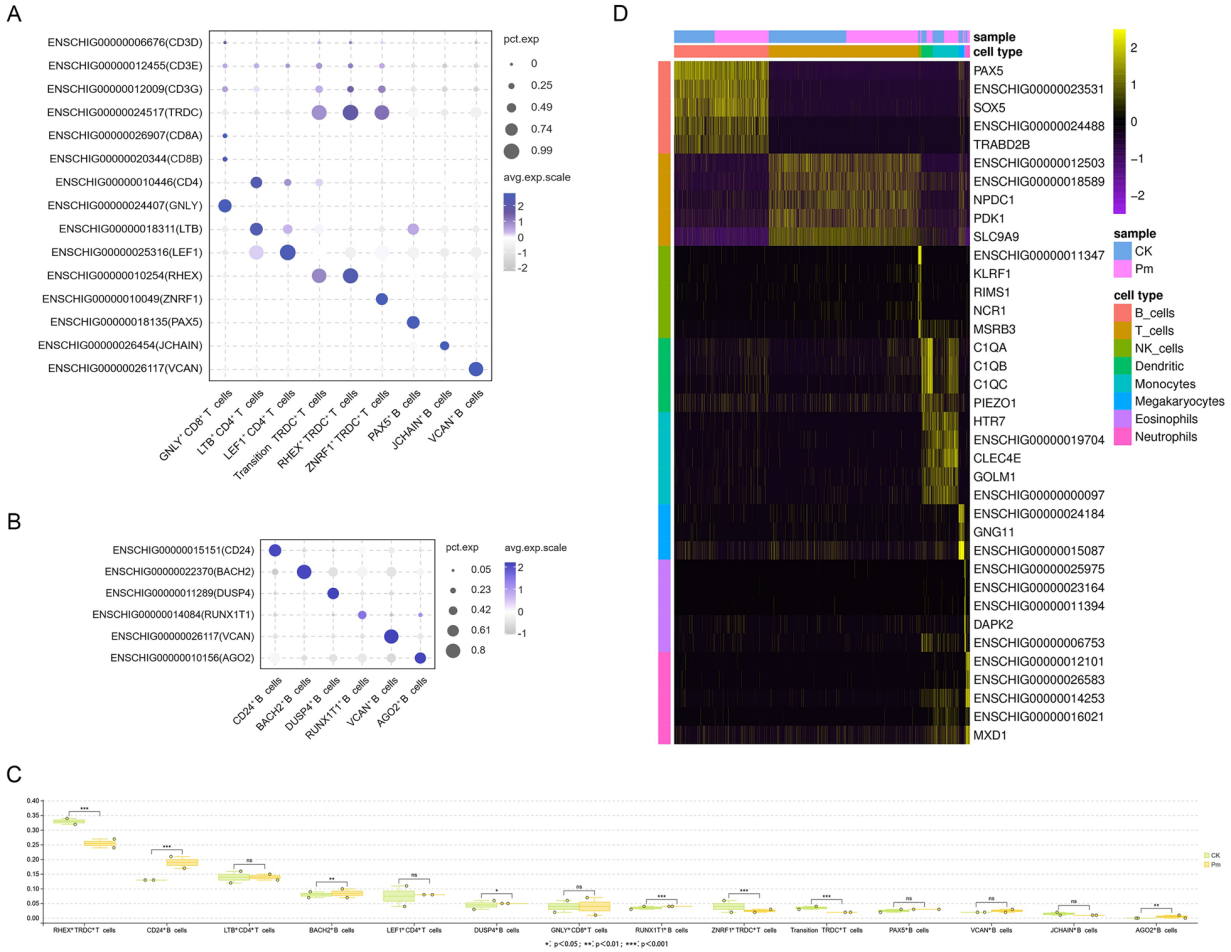
**T cell and B cell reclustering analysis and PBMC potential marker gene identifying in eight annotated goat cell populations**. **A** and **B** Bubble plot representing marker genes (*y*-axis) in each cluster (*x*-axis). Bubble size represents the proportion of genes expressed in the cluster; color represents the normalized value of the average gene expression in the cluster. **C** Chi-square test of each subset of T and B cell populations before and after infection with *P. multocida*. **D** Heat map showing the normalized expression of the top potential marker genes of each cell type in goat PBMCs.

To further elucidate the host response to *P. multocida* infection in goats, reclustering analyses were performed on B cell and T cell populations. These analyses identified eight distinct B cell subclusters and six T cell subclusters, which were named based on their highly expressed genes (Figures 5A and B). T cells were divided into CD4^+^ T cells, CD8^+^ T cells, and γδT cells (TRDC^+^ cells). Notaly, a transitional γδT cell subset (Transition TRDC^+^ T cells) was identified, characterized by co-expression of both *TRDC* and *CD4* genes without other exclusive highly expressed genes expression.

### Potential marker genes analysis

According to the results of cell annotation (Figure 4C) and the up-regulated expression genes of CK and Pm groups, the potential marker genes across distinct cellular subsets within goat PBMCs were screened. The results showed that B cells, T cells, NK cells, dendritic cells, monocytes, megakaryocytes, neutrophils and eosinophils had 55, 14, 26, 4, 12, 3, 7 and 13 potential marker genes, respectively (see Additional file 12). Cross-species comparative analysis of goat cellular potential markers across above eight cell populations through systematic interrogation of CellMarker and PanglaoDB databases revealed conserved B cell (*CD22*) and NK cell (*NCR1*, *IL2RB*) markers shared among goats, humans, and mice (Table 3). Heat map (Figure 5C) demonstrated high specificity of the top potential marker genes for six major cell types, while potential marker genes for dendritic cells and monocytes lacked sufficient discriminatory capacity to delineate these two immunophenotypically related populations.

**Table 3:** **Marker gene shared by goat, human and mouse**

Database	B cells	NK cells
PanglaoDB	MS4A1; CD22; CD79A; RASGRP3; PAX5; JCHAIN; BANK1; DERL3; FCHSD2; FCRLA; BLNK; BTLA; BCL11A	NCR1; IL2RB; CTSW; PRF1; ZBTB16
CellMarker	ENSCHIG00000014983; CD24; CD22	NCR1; IL2RB; CD226
Common genes	CD22	NCR1; IL2RB

### Differential expression analyses of peripheral blood cell types in goat

**Figure 6:**
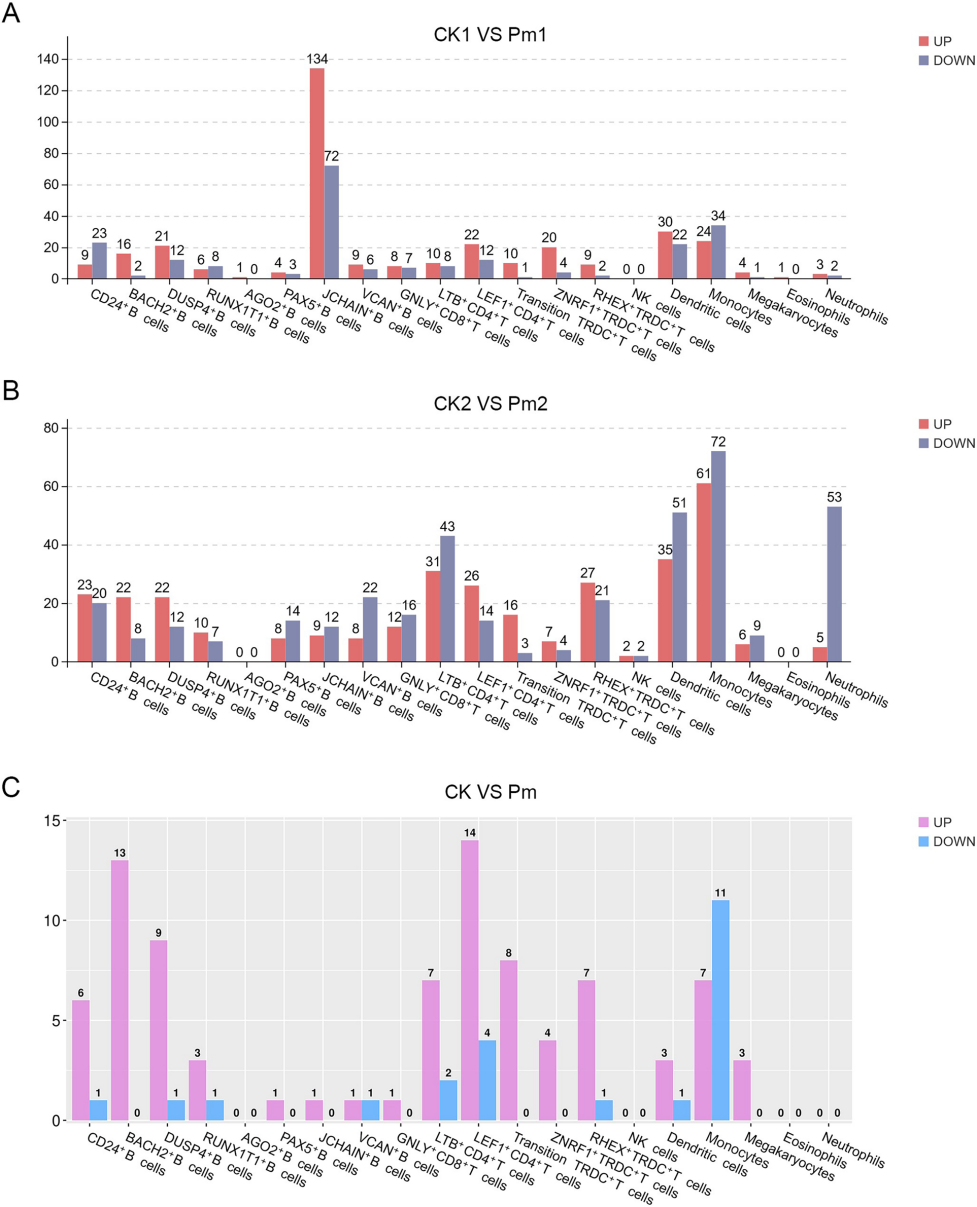
**DEGs screening across PBMC subpopulations from** ***P. multocida*-infected goats**. **A** and **B** Statistical analysis of up-/down-regulated DEGs across cell subpopulations in CK_1 versus Pm_1 (**A**) and CK_2 versus Pm_2 (**B**) groups. (**C**) Statistics of shared DEGs with consistent up-/down-regulation between comparative groups.

As shown in Figure 6, differential gene expression analysis between the CK_1 versus Pm_1 and CK_2 versus Pm_2 comparison groups identified 50 overlapping genes meeting stringent thresholds (|Log_2_(Fold Change)|≥ 1 and FDR < 0.05). Among these, 48 genes maintained concordant regulation directions (up- or down-regulated) in both comparisons, distributed across 16 cellular subpopulations (Figure 6 and Additional file 13). Notably, the majority of these shared DEGs exhibited up-regulation patterns. Three genes (*CXCR4*, *S100A12* and *S100A8*), demonstrated the most widespread cellular distribution, showing up-regulated expressed in 12, 9, and 8 distinct subpopulations, respectively.

### GO and KEGG analyses

**Figure 7:**
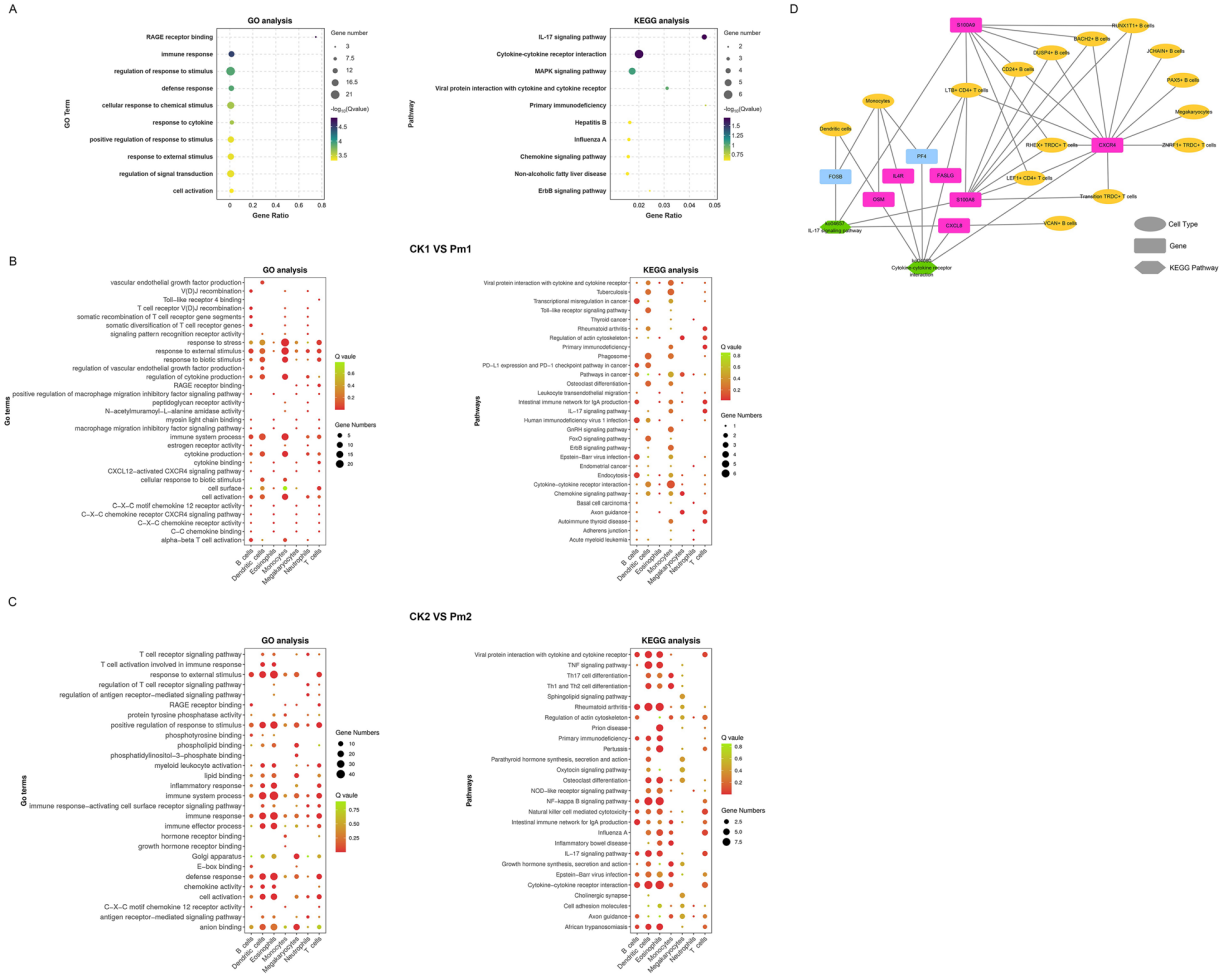
**Functional enrichment analysis of DEGs**. **A** Bubble plots showing GO (left) and KEGG (right) enrichment analyses of 48 common DEGs shared between the two comparison groups. **B** and **C** GO (left) and KEGG (right) enrichment bubble plots of DEGs across cell subpopulations in comparison group 1 (**B**) and comparison group 2 (**C**), respectively. **D** Network diagram illustrating significantly enriched KEGG pathways, associated genes, and cell subpopulations for common DEGs shared between both comparison groups. Up-regulated DEGs are indicated in magenta, while down-regulated DEGs are shown in blue.

Functional annotation of 48 DEGs through GO and KEGG pathway analyses revealed immune-related biological processes and signaling pathways (Figure 7A, Additional files 14 and 15). Specifically, 14 and 21 genes were significantly enriched in immune response and regulation of response to stimulus GO terms. Pathway analysis identified four genes enriched in the IL-17 signaling pathway and six genes participating in cytokine–cytokine receptor interaction with both pathways showing statistical significance (FDR < 0.05). Further analysis revealed that *CXCR4* was up-regulated across 12 distinct cell types, whereas *IL4R*, *FASLG*, and *CXCL8* exhibited up-regulation specifically in monocytes and LTB^+^ CD4^+^ T cells (Figure 7D).

GO and KEGG enrichment analyses were conducted on DEGs from two goats in CK and Pm groups, respectively. The results showed (Figure 7B) that in CK_1 versus Pm_1 group, all cells enriched GO term was response to external stimulus, while in CK_2 versus Pm_2 group (Figure 7C), all cells enriched GO term was positive regulation of response to stimulus, immune system process, immune response, and defense response. In the two comparison groups, the cell subpopulations were both significantly enriched to four GO terms (GO:0009605, GO:0050786, GO:0002376, GO:0001775) (see Additional file 16). Notably, in the CK_1 versus Pm_1 comparison group, monocytes exhibited the highest number of DEGs (21 genes) associated with the significantly enriched GO term response to stress (GO:0006950) across nine major cell populations. In the CK_2 versus Pm_2 group, monocytes remained predominant (40 genes), alongside dendritic cells as a distinct contributor (27 genes), with both cell subsets showing significant enrichment in immune system process (GO:0002376).

KEGG enrichment analysis showed monocyte-associated genes in the CK_1 versus Pm_1 group were predominantly enriched in the cytokine–cytokine receptor interaction pathway (Figure 7B). In CK_2 versus Pm_2 group, most genes were enriched in TNF signaling pathway, rheumatoid arthritis, NF-kappa B signaling pathway, and cytokine–cytokine receptor interaction pathway of dendritic cells and eosinophils (Figure 7C). The two comparison groups shared six identical significantly enriched KEGG pathways (ko04672, ko04061, ko04060, ko05340, ko04657, ko05323) (see Additional file 17).

Enrichment analysis of B and T cell subpopulations revealed that only JCHAIN^+^ B cells exhibited a greater number of DEGs compared with other subsets in the CK_1 versus Pm_1 group (see Additional files 16, 17 and 18). Multiple cell subsets in this comparison group showed significant enrichment for the intestinal immune network for IgA production (ko04672) and IL-17 signaling pathway (ko04657). In the CK_2 versus Pm_2 group, two CD4^+^ T cell subpopulations were enriched with more DEGs. Besides the above two pathways (ko04672 and ko04657), B cell receptor signaling pathway (ko04662) was also significantly enriched in multiple cell subsets. In total, 10 GO terms (GO:0009605, GO:0050786, GO:0008120, GO:0038147, GO:0008138, GO:0005509, GO:0035662, GO:0003779, GO:0002429, GO:0002768) and 5 KEGG pathways (ko05340, ko04672, ko04657, ko04360, ko04380) were significantly enriched in both comparison groups.

### Gene set score

**Figure 8:**
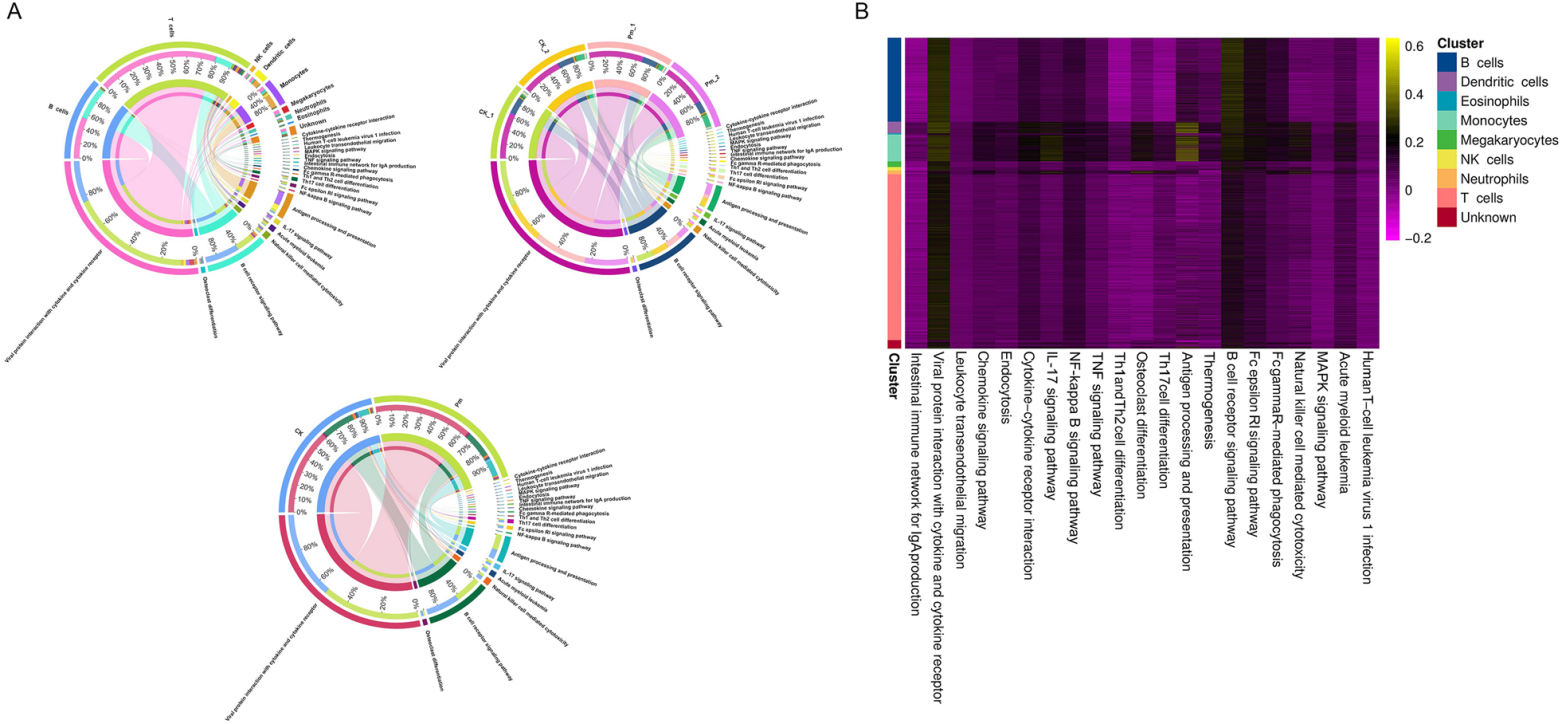
**The gene sets of the 21 KEGG pathways were scored**. **A** Circular plot displays gene set scores at three hierarchical levels including cell subpopulation, individual sample and study group (CK and Pm groups). **B** Heatmap of scoring gene sets for each cell subpopulation.

Gene set scoring analysis was performed on 21 selected KEGG pathways using pathway-associated genes. The results (Figure 8) showed B cells primarily engaged in the viral protein interaction with cytokine and cytokine receptor and B cell receptor signaling pathway, whereas dendritic cells and monocytes predominantly contributed to antigen processing and presentation. Similar scoring patterns between the CK_1 versus Pm_1 and CK_2 versus Pm_2 groups indicated consistent sample processing. The B cell receptor signaling pathway exhibited the highest scores in B cells compared with other cell subpopulations. In T cells, viral protein interaction with cytokine and cytokine receptor showed the highest pathway ranking, while dendritic cells displayed the maximal scores for this pathway (see Additional file 19A). Intergroup difference analysis demonstrated significant post-infection score increases in viral protein interaction with cytokine and cytokine receptor, thermogenesis, IL-17 signaling pathway, cytokine–cytokine receptor interaction, and antigen processing and presentation pathways. Conversely, MAPK signaling pathway, natural killer cell mediated cytotoxicity, NF-kappa B signaling pathway, and Th17 cell differentiation pathways displayed marked score reductions (see Additional file 19B).

### Cell trajectory analysis

**Figure 9:**
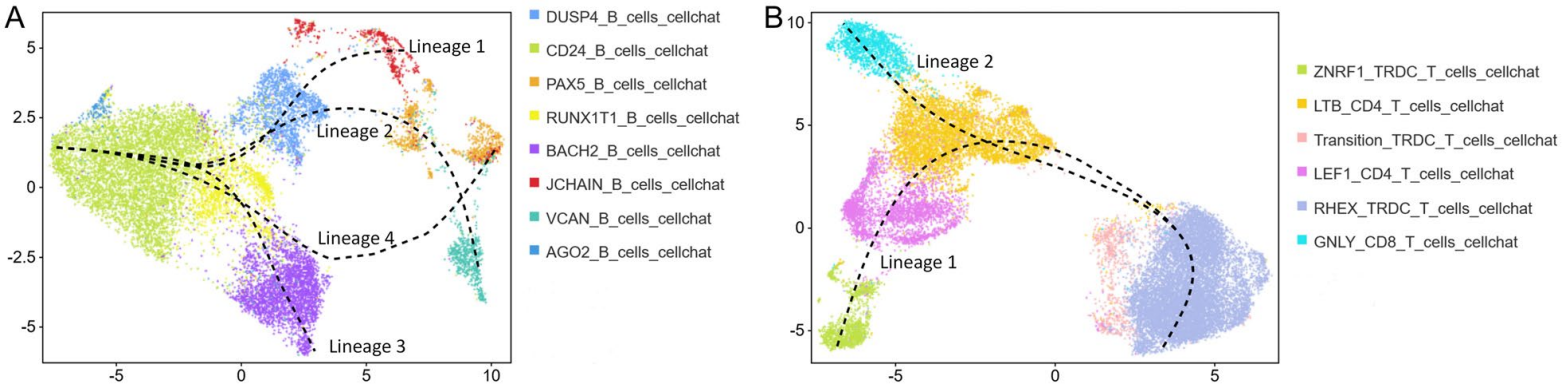
**Cell trajectory analysis of B cells (A) and T cells (B) in goat PBMCs**.

**Figure 10:**
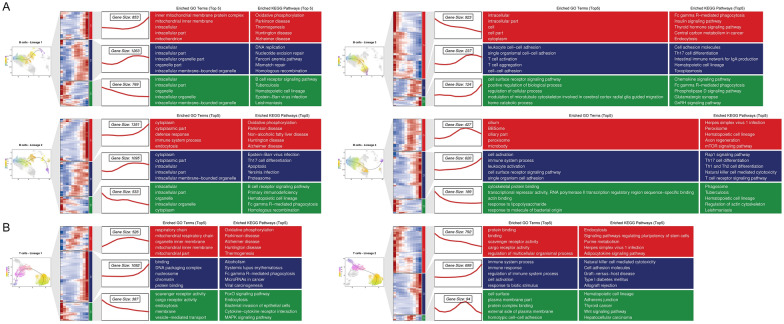
**Functional enrichment analysis of DEGs in B (A) and T cell (B) lineages.** The UMAP dimensionality reduction map of the differentiated lineages is shown on the left. The middle shows the clustering results of differentially expressed genes. The top five GO terms and KEGG pathways are shown on the right.

Trajectory analyses of B and T cells revealed four and two distinct differentiation lineages, respectively (Figure 9). Cluster analysis of DEGs for each lineage grouped the DEGs into three clusters per lineage (see Additional file 20). Within the B cell differentiation lineage (Figure 10A), DEGs in Lineage 1 and Lineage 2 were predominantly enriched in GO terms related to cellular components. Notably, Cluster 1 (C1) of Lineage 2 also showed enrichment in defense response, immune system process, and endocytosis GO terms. C2 of Lineage 3 was enriched in GO terms associated with T cell activation, aggregation, and cell adhesion. C1 of Lineage 4, exhibiting an expression trend consistent with C2 of Lineage 3, was primarily enriched in cilia and microbodies related GO Terms. Furthermore, genes displaying an up-regulated expression trend in Lineage 4 were mainly enriched in immune-related GO terms. Importantly, during the differentiation of CD24^+^ B cells into PAX5^+^ B cells, genes with a down-regulated trend were enriched in the response to lipopolysaccharide and molecule of bacterial origin GO terms. KEGG enrichment analysis demonstrated that DEGs from Lineages 2, 3, and 4, except Lineage 1, were enriched in Th17 cell differentiation. DEGs with an up-regulated trend in Lineage 4 were mainly enriched in T cells related KEGG pathways. Additionally, down-regulated DEGs in Lineage 2 and 3, along with up-regulated DEGs in Lineage 3, were enriched in the Fc gamma R-mediated phagocytosis pathway, suggesting a potential role of this pathway in the host’s clearance of *P. multocida* during early infection. Analysis of DEGs in the two T cell lineages (Figure 10B) indicated that down-regulated genes were enriched in scavenger and cargo receptor activity GO terms, while up-regulated genes in Lineage 2 were enriched primarily in immunity-related GO terms. KEGG analysis showed that up-regulated genes in Lineage 1 and 2 were enriched in Fc gamma R-mediated phagocytosis and natural killer cell mediated cytotoxicity pathways, respectively. For down-regulated genes, Lineage 1 showed enrichment in FoxO and MAPK signaling pathways, and Lineage 2 in adipocytokine signaling pathway. These pathways play crucial roles in immunity.

### Ligand-receptor intercellular communication network analysis

**Figure 11:**
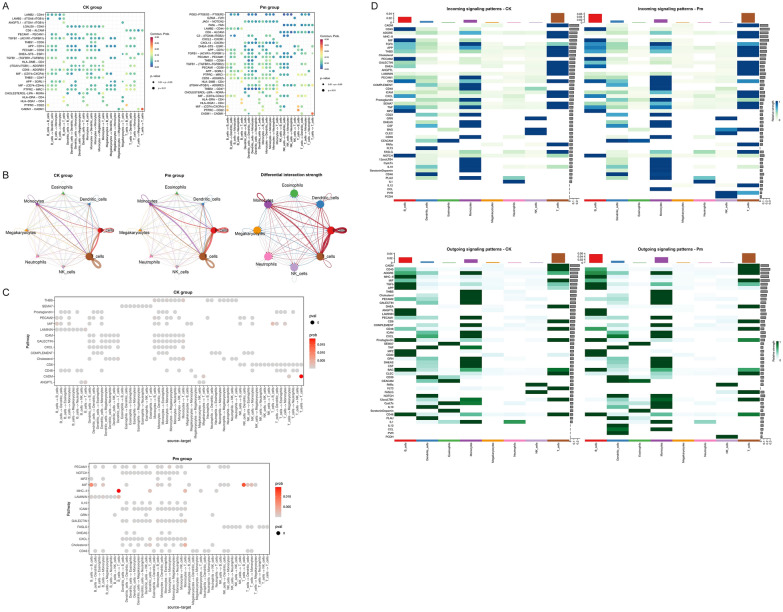
**Intercellular communication analysis in goat PBMCs pre- and post-*P. multocida*** **infection**. **A** Dot diagrams showing the number of ligand–receptor pairs and the communication probability between ligand cells (top 5) and receptor cells (top 5) in CK and Pm groups, respectively. **B** Network diagram showing the number of ligand–receptor pairs and communication probability information in eight cell populations. The size of the peripheral circle is proportional to the ratio of the number of intercellular ligand–receptor pairs. The thickness of the line indicates the strength of the change in communication. In the right network diagram, the blue line indicates that the communication of the CK group is stronger, and the red line indicates that the communication of the Pm group is stronger. **C** Bubble charts displaying the top 15 cell populations ligand–receptor pairs with highest communication probability for each of the 15 most statistically significant signaling pathways based on minimal *P*-values in CK and Pm groups, respectively. **D** Heat maps displaying the communication patterns of eight cell populations in PBMCs of uninfected or infected goats with ***P. multocida***. The columns on the upper and right sides are the accumulation of longitudinal and transverse strength.

To investigate cellular interactions within the blood, significant ligand–receptor pairs in the CK and Pm groups were predicted using CellChat, and differences in communication levels between the groups were analyzed (see Additional files 21 and 22). The results (Figure 11A) demonstrated that intercellular communication was conspicuously enhanced after infection. For instance, the communication probability of ligand–receptor pairs such as PTPRC-CD22, MIF-(CD74 + CXCR4), HLA-DQA1-CD4, HLA-DRA-CD4, and MIF- (CD74 + CD44) were significantly higher in the Pm group compared with the CK group. Notably, CADM1—CADM1 was the ligand–receptor pair exhibiting the highest communication probability in T cell self-communication, irrespective of *P. multocida* infection status. Communication analysis of T cell subpopulations further indicated that CADM1—CADM1 primarily functions in RHEX^+^ TRDC^+^ T cells and Transition TRDC^+^ T cells (see Additional file 23B). Transition TRDC^+^ T cells represent transitional intermediates between RHEX^+^ TRDC^+^ T cells and other T cell subpopulations (Figure 10B), suggesting that the CADM1—CADM1 ligand–receptor pair may play a key role in this process. At the cellular level, significant post-infection increases were observed not only in B cell self-communication but also in B cell—T cell communication and in monocytes communication with both B cells and T cells (Figure 11B, Additional file 23A and C). While communication involving NK cells and other subpopulations was inconspicuous prior to infection, the expression abundance of ligand–receptor pairs on this subpopulation increased post-infection. Subpopulation analysis revealed that CD24^+^ B cells, acting as source cells, exhibited significantly enhanced communication probability with DUSP4^+^ B cells, JCHAIN^+^ B cells, and BACH2^+^ B cells after infection. And BACH2^+^ B cells → AGO2^+^ B cells and AGO2^+^ B cells ↔ AGO2^+^ B cells produced communication signals (see Additional file 23D). Among T cell subpopulations, the communication probability between GNLY^+^ CD8^+^ T cells → LTB^+^ CD4^+^ T cells\Transition TRDC^+^ T cells, and Transition TRDC^+^ T cells → Transition TRDC^+^ T cells was enhanced (see Additional file 23E). Furthermore, in the communication analysis between the subpopulations of B, T cells and monocytes (see Additional file 23F), the communication probabilities between CD24^+^ B cells and LTB^+^ CD4^+^ T cells, RHEX^+^ TRDC^+^ T cells and monocytes, as well as between LTB^+^ CD4^+^ T cells and BACH2^+^ B cells, were all significantly enhanced in the Pm group. Moreover, monocytes, as source cells, exhibited strengthened self-communication probability and enhanced communication with LTB^+^ CD4^+^ T cells and RHEX^+^ TRDC^+^ T cells post-infection.

At the level of signaling pathways (Figure 11C), the CADM signaling pathway within T cells exhibited the highest communication probability prior to infection. However, following *P. multocida* infection, communication probability was significantly enhanced in the MIF signaling pathway (within B cells and between T and B cells) and in the MHC-II signaling pathway (between B and T cells) (Figure 11C, Additional file 22). Interestingly, subpopulation analysis revealed that the communication probability intensity of the MHC-II signaling pathway exhibited enhanced levels in T cell subpopulations post-infection, whereas B cell subpopulations showed heterogeneous changes (not uniformly enhanced) compared with pre-infection states. (see Additional file 23G and H). In addition, the communication probability of signaling pathways between monocytes and T cells was enhanced (Figure 11C), with the THBS signaling pathway showing the most pronounced increase (see Additional file 23I). Heatmap indicated that B cells displayed the most significant communication enhancement post-infection. Monocytes exhibited more pronounced alterations in outgoing versus incoming signals, whereas T cells demonstrated the strongest incoming and outgoing signal intensities across both CK and Pm groups. B cells modulated preexisting signaling pathways via enhancement or suppression, while monocytes activated CCL, IL10 and NOTCH pathways and T cells activated BAG, IL12, PVR, and Prostaglandin pathways after infection (Figure 11D). Subpopulation analysis suggested that AGO2^+^ B cells represent an inactive subtype. Additionally, accumulated signal intensity in T cell subpopulations showed negligible change post-infection (see Additional file 23J and K).

### Integrated analysis of RNA-seq and scRNA-seq

**Figure 12:**
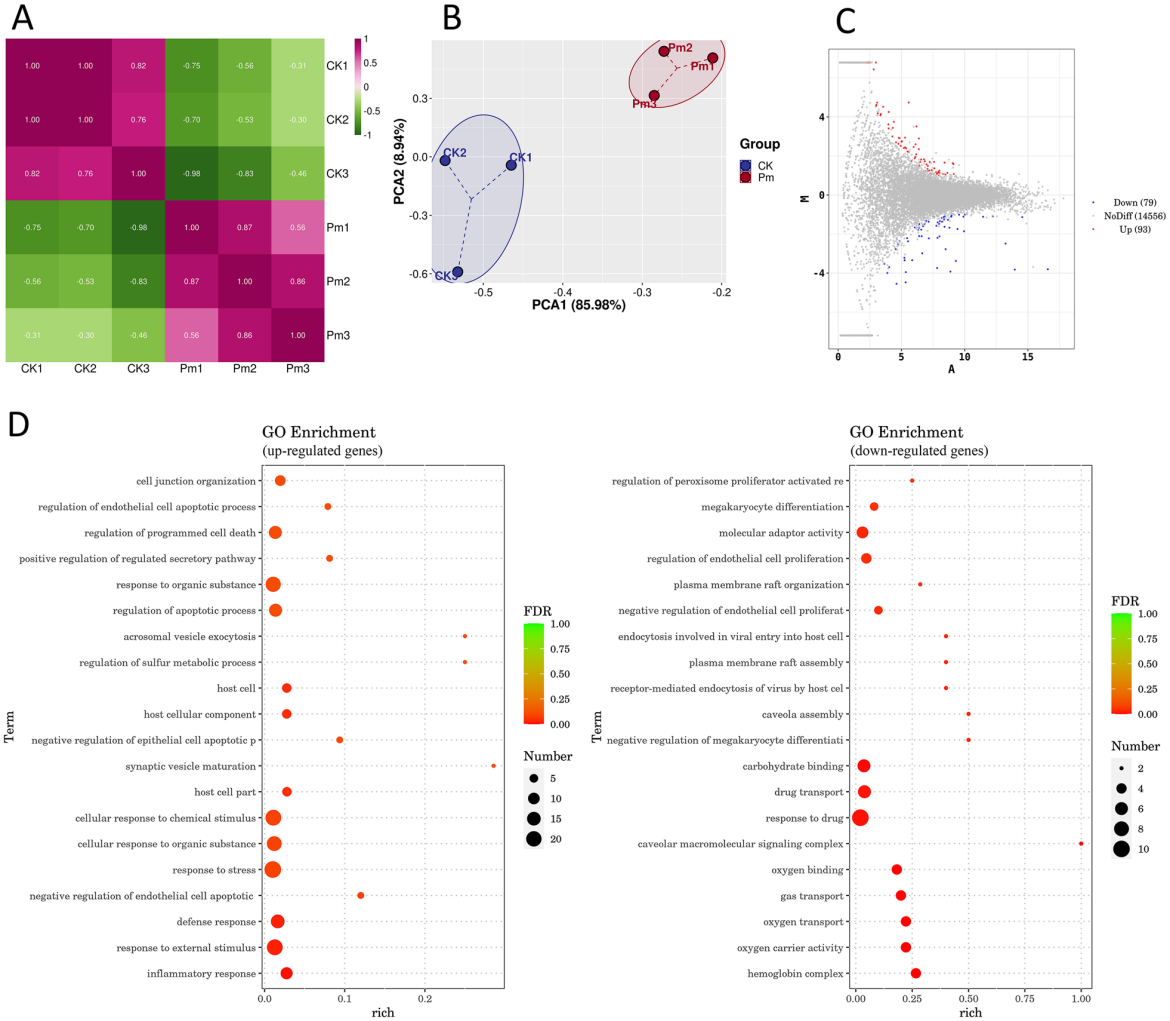

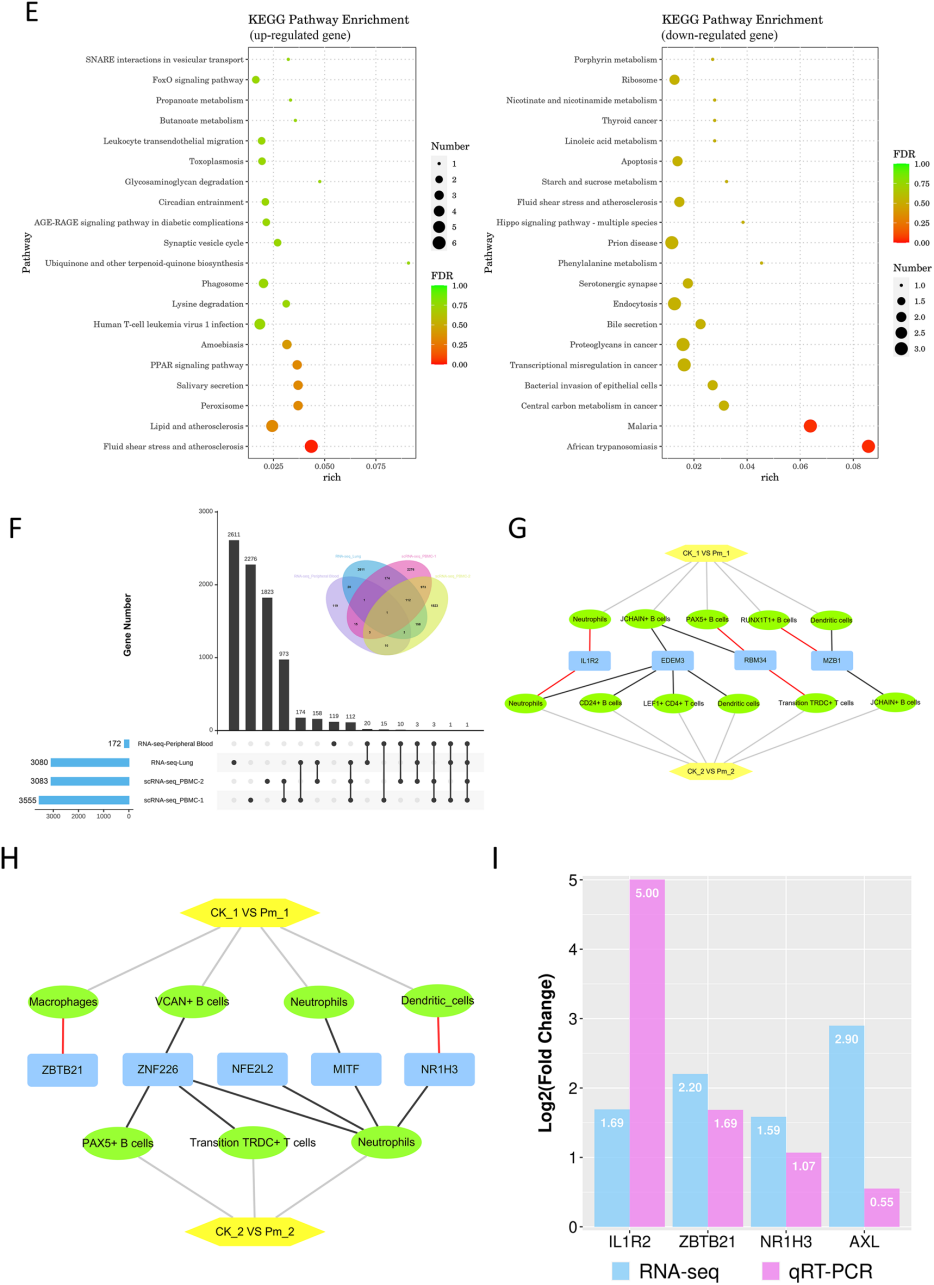
**Integrated analysis of RNA-seq and PBMCs scRNA-seq data from** ***P. multocida*-infected goats**. **A** Heatmap of sample correlation analysis for peripheral blood RNA-seq. **B** PCA of peripheral blood RNA-seq samples. **C** MA plot displaying DEGs screened from peripheral blood RNA-seq data. **D** and **E** Bubble plots of GO (**D**) and KEGG (**E**) enrichment analyses for DEGs from peripheral blood RNA-seq data. **F** UpSet plot of DEGs across four datasets from ***P. multocida***-infected goats at 24 h post-infection. **G** Expression profiles of *IL1R2* (shared across all four sequencing datasets) and *RBM34*/*EDEM3*/*MZB1* (common to three blood RNA-seq datasets) in various cell subpopulations between comparison groups. Red lines represent up-regulated expression; black lines indicate down-regulation. **H** Expression profiles of differentially expressed transcription factors shared between peripheral blood RNA-seq and PBMC scRNA-seq datasets are displayed across cell subpopulations in comparative groups. **I** qRT-PCR validation of four randomly selected up-regulated DEGs from peripheral blood RNA-seq data.

RNA sequencing of peripheral blood from goats pre- and post-*P. multocida* infection generated high-quality data, with Q20% and Q30% values exceeding 94% and alignment rates to the goat reference genome surpassing 96% for all samples (Table 4). Sample correlation heatmap (Figure 12A) and principal component analysis (PCA) (Figure 12B) confirmed high intragroup reproducibility. Differentially expression analysis identified 172 DEGs (93 up-regulated, 79 down-regulated). Four randomly selected up-regulated DEGs (*IL1R2*, *ZBTB21*, *NR1H3*, *AXL*) were validated by qRT-PCR. Results demonstrated consistent expression trends with sequencing data (Figure 12I), confirming the reliability of the RNA-seq dataset.

**Table 4:** **RNA sequencing data quality control and reference genome comparison results**

Projects	CK1	CK2	CK3	Pm1	Pm2	Pm3
Raw reads	58,978,244	48,272,404	47,000,998	78,657,176	39,765,676	45,525,088
Clean reads	57,755,308	47,141,544	45,922,518	77,087,514	38,924,898	44,559,848
Q20%	97.09	97.1	97.18	97.07	96.79	96.81
Q30%	94.89	94.84	94.94	94.84	94.34	94.4
Total mapped	55,725,940 (96.49%)	45,525,570 (96.57%)	44,552,807 (97.02%)	74,863,473 (97.11%)	37,643,767 (96.71%)	43,226,474 (97.01%)
Multiple mapped	2,951,870 (5.30%)	2,713,087 (5.96%)	2,748,202 (6.17%)	3,395,498 (4.54%)	2,131,210 (5.66%)	2,260,588 (5.23%)
Uniquely mapped	52,774,070 (94.70%)	42,812,483 (94.04%)	41,804,605 (93.83%)	71,467,975 (95.46%)	35,512,557 (94.34%)	40,965,886 (94.77%)
Mapped_to_Gene	40,749,725 (77.22%)	33,377,351 (77.96%)	32,921,006 (78.75%)	53,401,211 (74.72%)	27,739,909 (78.11%)	31,747,837 (77.50%)
Mapped_to_InterGene	12,024,345 (22.78%)	9,435,132 (22.04%)	8,883,599 (21.25%)	18,066,764 (25.28%)	7,772,648 (21.89%)	9,218,049 (22.50%)
Mapped_to_Exon	36,938,146 (90.65%)	30,504,471 (91.39%)	29,153,768 (88.56%)	47,363,012 (88.69%)	25,234,058 (90.97%)	28,888,393 (90.99%)

GO enrichment analysis revealed up-regulated DEGs predominantly enriched in response to external stimuli and defense, whereas down-regulated DEGs primarily associated with substance transport (Figure 12D). Directed acyclic graph analysis further demonstrated functional enrichment of up-regulated DEGs in inflammatory responses, such as modulation of enzymatic activities, while down-regulated DEGs showed association with oxygen binding processes within hemoglobin complexes for oxygen transport (see Additional file 24A). KEGG enrichment analysis demonstrated significantly enrichment of up-regulated DEGs in the fluid shear stress and atherosclerosis pathway, while down-regulated DEGs were primarily enriched in parasitic infectious diseases pathways (malaria and African trypanosomiasis) (Figure 12E). Metabolic mapping of these three enriched pathways indicated potential involvement of up-regulated genes in adhesion processes, with down-regulated genes implicated in killing pathogenic microorganisms, immunosuppression, and coagulation dysfunction (see Additional file 24B).

DEGs from lung RNA-seq data (1-day post-*P. multocida* infection) in our previous study and scRNA-seq data from this experiment were re-screened using thresholds of |Log_2_(Fold Change)|> 1 and *P*-value < 0.05. These re-screened DEGs were compared with the aforementioned 172 DEGs to identify the genes with consistently regulated genes. Analysis revealed *IL1R2* as the only up-regulated DEG across all four datasets (Figure 12F and Additional file 25). Among three blood datasets, the commonly up-regulated *RBM34* (*ENSCHIG00000020834*) and down-regulated *EDEM3* and *MZB1* were identified. These four genes were differential expression in five cell types in CK_1 versus Pm_1 and six cell types in CK_2 versus Pm_2 comparisons (Figure 12G). Expression trends of *IL1R2* and *EDEM3* across five cell types aligned with RNA-seq data, while *RBM34* and *MZB1* showed concordant regulation with peripheral blood RNA-seq in PAX5^+^ B cells, Transition TRDC^+^ T cells, dendritic cells, and JCHAIN^+^ B cells, but divergent trends in two other cell types. Statistical analysis of differentially expressed transcription factors in peripheral blood RNA-seq (Table 5) identified 13 differentially expressed transcription factors classified into eight families (5 up-regulated, 8 down-regulated). Comparison of these transcription factors with scRNA-seq DEGs demonstrated concordant up-regulation of *ZBTB21* and *NR1H3* in monocytes and dendritic cells in the CK_1 versus Pm_1 comparison group with RNA-seq data. Conversely, all other transcription factors exhibited uniformly down-regulated expression across four cell types with discordant patterns (Figure 12H).

**Table 5:** **Differential transcription factor statistics**

Gene_ID	Gene_name	Family	Description	Trend
ENSCHIG00000022111	MITF	bHLH	Melanocyte inducing transcription factor	Up
ENSCHIG00000025552	SOX18	HMG	SRY-box transcription factor 18	Down
ENSCHIG00000011281	SMAD1	MH1	SMAD family member 1	Up
ENSCHIG00000024210	PURG	Others	Purine rich element binding protein G	Down
ENSCHIG00000014331	–	Others	YEATS domain containing 4	Up
ENSCHIG00000024067	CEBPE	TF_bZIP	CCAAT enhancer binding protein epsilon	Down
ENSCHIG00000026055	NFE2L2	TF_bZIP	Nuclear factor, erythroid 2 like 2	Up
ENSCHIG00000013751	NR1H3	THR-like	Nuclear receptor subfamily 1 group H member 3	Up
ENSCHIG00000022184	ZBTB21	ZBTB	Zinc finger and BTB domain containing 21	Up
ENSCHIG00000007068	ZNF391	zf-C2H2	Zinc finger protein 391	Up
ENSCHIG00000015070	ZNF226	zf-C2H2	Zinc finger protein 226	Up
ENSCHIG00000017383	ZNF304	zf-C2H2	Zinc finger protein 304	Down
ENSCHIG00000008990	GATA1	zf-GATA	GATA binding protein 1	Down

## Discussion

The blood harbors a large population of immune cells that constitutively circulate throughout the body and remain poised to defend against invading pathogenic microorganisms. Concurrently, this circulation enables the rapid transport of immune cells via the bloodstream to tissues and organs to resist pathogens. However, studies investigating the interaction between goat PBMCs and pathogens using scRNA-seq technology remain scarce. In this study, scRNA-seq was employed to analyze immune cell composition within goat PBMCs before and after *P. multocida* infection, thereby exploring the role of circulating immune cells in host immune responses.

Prior to *P. multocida* infection, five candidate goats underwent clinically examinations to ensure baseline health. A preliminary experiment with goat No. 2 confirmed that the designated challenge dose reliably induced pulmonary lesions within 24 h. Immune responses exhibit substantial heterogeneity across individuals due to intrinsic resistance and environmental factors. To mitigate the impact of individual variation on the experimental data, PBMCs of CK and Pm groups were collected from the same goats before and after *P.multocida* infection, with two biological replicates per treatment. However, integrated assessment of health monitoring, pulmonary lesion severity, RPA-LFD detection results, and statistical analysis of DEGs across PBMC subsets revealed a more pronounced immune response in goat No. 3. This indicated deeper infection intensity and comparatively greater resistance of immune cells to *P. multocida* relative to goat No. 5. Comparative analysis of RPA-LFD and HE staining demonstrated undetectable *P. multocida* in the spleen of goat No. 5, whereas goat No. 3 exhibited only a faint detection signal. This may suggest incomplete splenic colonization by *P. multocida* in goat No. 5 at the 24 h timepoint. Moreover, RPA-LFD detection of peripheral blood showed prominent signals in goat No. 5 post-infection, but minimal *P. multocida* presence in goat No. 3. This further supports the hypothesis that *P. multocida* remained predominantly circulating in goat No. 5’s bloodstream without achieving full tissue colonization. No bacteria were detected in the lesioned left lung tissue of goat No. 3. Histological examination revealed severe pulmonary consolidation with extensive granulocyte and macrophage infiltration in this tissue. GO enrichment analysis suggested most cells in goat No. 5 were actively responding to external stimuli, whereas goat No. 3 had initiated immune defense mechanisms. Collectively, these observations indicate that *P. multocida* in the lesioned lung tissue of goat No. 3 may have been eliminated prior to detection.

Owing to the absence of well-defined marker genes for goat immune cell classification, B cell and T cell subpopulations were annotated using specifically highly expressed genes. *PAX5*, *CD24*, and *JCHAIN* were identified as potential B cells markers (see Additional file 12). *PAX5* is a key B cell lineage differentiation factor that restricts lymphoid progenitor commitment to the B cell lineage. Genetic ablation of *PAX5* drives reversion of B lymphocytes to undifferentiated progenitors, and *PAX5* expression is absent in plasma cells [45]. CD24 functions as an early differentiation marker during B cell development [37]. Furthermore, DUSP4^+^ B cells annotated in this study may represent atypical memory (AtM) B cells capable of differentiating into antibody secreting cells (ASCs) [38], while *JCHAIN* constitutes a canonical plasma cell marker [39]. Differentiation trajectory analysis (Figures 9 and 10) revealed two distinct lineages (Lineage 1 and Lineage 2) originating from DUSP4^+^ B cells that align with the abovementioned functional characteristics, supporting pseudotime analysis reliability. These observations further suggest VCAN^+^ B cells may represent an ASC population distinct from JCHAIN^+^ B cells. Furthermore, it is noteworthy that while *CD163* and *CD86* are generally regarded as marker genes for macrophages, cells can only be strictly defined as macrophages upon tissue residency. Since these markers are also associated with monocytes, the corresponding cell populations were annotated as monocytes. The detection of these gene expressions in monocytes indicated their differentiation toward macrophages and denoted an activated immune state, which aligns with our findings.

A comparative analysis of RBPT and scRNA-seq data demonstrated a consistent increase in neutrophil and monocyte levels following infection across both analytical platforms (Figure 1A and Table 2). This agreement strongly supports the central role of these innate immune cells in early pathogen control. The lymphocyte compartment presented a more complex pattern. RBPT indicated a relatively stable total lymphocyte count in peripheral blood, whereas scRNA-seq further delineated a marked compensatory dynamic characterized by a significant expansion of B cells from 27.48% to 34.57% accompanied by a concurrent decrease in T cells from 52.82% to 45.86%. These findings collectively indicate that the apparent stability in total lymphocyte count masks a substantial and rapid reorganization of the immune landscape.

Analysis of cellular communication revealed that following *P. multocida* infection, 12 significantly enhanced differential ligand–receptor pairs were identified in the MHC-II signaling pathway, specifically for interactions from dendritic cells\monocytes\B cells → T cells. Within the MHC-I signaling pathway, the receptor cells were exclusively CD8^+^ T cells, and all differential ligand–receptor pairs exhibited a weakening trend. Gene set scoring results further indicated that dendritic cells and monocytes primarily function in antigen processing and presentation. These findings suggest that *P. multocida*-derived exogenous antigens are likely being internalized, processed, and presented to T cells via the MHC-II pathway by dendritic cells or monocytes. Additionally, given their expression of MHC II molecules and co-stimulatory molecules, B cells are also capable of presenting *P. multocida*-derived protein antigens to T cells. It is well established that γδT cells can directly recognize and bind antigens independently of MHC molecules and antigen-presenting cells. At 24 h post-infection, all three subsets of γδT cells exhibited a highly significantly decrease, while CD4^+^ T cells and CD8^+^ T cells remained relatively stable. This observed sharp decline in γδT cells is likely attributed to their recruitment to the mucosal tissues of the lungs, where they may perform functions such as recognizing and eliminating infected cells. Given the relatively short duration of infection, this process may not yet have elicited a robust adaptive immune response. Conversely, five B cell subsets showed a significant increase, particularly CD24^+^ B cells and RUNX1T1^+^ B cells (Figure 5C). This may be due to the activation of humoral immunity, which promotes B cell proliferation to facilitate antibody production against the pathogen. These observations indicate that the initial host response to *P. multocida* infection is predominantly non-specific immunity. Fu et al. reported that RNA-seq analysis of goat peripheral blood lymphocytes infected with type D *P. multocida* identified DEGs significantly enriched in the IL-17 signaling pathway and cytokine–cytokine receptor interactions [46], consistent with our findings. Therefore, it is speculated that among the multiple immune defense pathways activated during the initial host response to *P. multocida* infection, one crucial pathway involves γδT cells producing the pro-inflammatory cytokine IL-17 in response to exogenous stimuli [47]. This IL-17-mediated response potentially induces chemokine expression, resulting in increased neutrophils (Figure 4E) that capture and eliminate *P. multocida,* thereby controlling bacterial infection within the host. Although the chemokine *CXCL12* was not among the screened 48 DGEs (see Additional file 13), its specific receptor *CXCR4* was an up-regulated DGE in 12 cell subsets. Furthermore, the chemokine *CXCL8* was an up-regulated DGE in JCHAIN^+^ B cells. Consequently, *CXCL8* and *CXCL12* may be the IL17-induced chemokines. Additionally, Wang et al. observed a significant reduction in the abundance of NK cells and γδT cells in severe tuberculosis caused by *Mycobacterium tuberculosis*, suggesting lymphocytopenia may be a prominent feature of severe disease [48]. They proposed that systematically up-regulated *S100A12* in peripheral blood monocytes may contribute to the cytokine storm in severe patients. Data from our study similarly demonstrated decreased abundance of both NK cells and γδT cells (Figures 4E and 5C). However, in contrast to their findings, *S100A12* was primarily up-regulated in B cells, T cells and megakaryocytes in our research (see Additional file 13).

Integrated analysis of RNA-seq and scRNA-seq data identified potentially critical genes during the initial phase of *P. multocida* infection. IL1R2 serves as a key mediator of innate immunity and inflammation, regulating both protective and pathological immune responses through modulation of cellular activities including proliferation, differentiation, and survival or apoptosis [49]. Sun et al. demonstrated that IL33 induces high-level IL1R2 surface expression on neutrophils, in contrast to the inability of LPS to elicit this response, and designated IL1R2 as a biological marker for IL33-induced neutrophils [50]. In this study, *IL1R2* was the only consistently up-regulated gene across all four *P. multocida*-infected datasets at 24 h post-infection, with expression exclusively elevated in neutrophils. This expression pattern suggests these neutrophils may represent an IL33-induced subpopulation. Furthermore, according to reports, *IL1R2* may serve as a peripheral blood diagnostic biomarker for acute myocardial infarction in early-stage type 1 diabetes, demonstrating positive correlation with neutrophil levels. This suggests neutrophil-derived *IL1R2* in peripheral blood as a potential early detection marker for *P. multocida* infection. Analysis of three independent blood sequencing datasets identified *RBM34*, *EDEM3*, and *MZB1* as shared DEGs. RBM34, an RNA-binding protein, was up-regulated in PAX5^+^ B cells and Transition TRDC^+^ T cells (Figure 12G). Pseudotime analysis revealed neither cell subpopulation belongs to terminally differentiated cells (Figures 9 and 10). These observations suggest *RBM34* may regulate mRNA processing of immune-related genes in these cells, thereby modulating their activation, proliferation, and differentiation to ensure the host generates and maintains highly efficient immune responses against *P. multocida*. EDEM3 primarily facilitates degradation of misfolded glycoproteins in the endoplasmic reticulum [51]. MZB1, an endoplasmic reticulum-resident protein, shows significant up-regulation during plasma cell differentiation [52]. Down-regulation of both genes in canonical plasma cells (JCHAIN^+^ B cells) may impair proper antibody folding and secretion, compromising the antibody-secreting capacity of these cells, specifically for polymeric IgA and IgM. Accumulation of misfolded proteins could further affect cellular viability and function. However, in this study, JCHAIN^+^ B cell abundance showed no significant change post-infection (Figure 5C), likely owing to the brief infection duration.

This study presents the first application of scRNA-seq technology for high-throughput sequencing of circulating immune cells (PBMCs) in goats infected with *P. multocida*. Eight distinct PBMC populations were resolved, with B cells and monocytes exhibiting significant expansion post-infection, while the number of T cells and megakaryocytes showed marked reduction. Potential marker genes were screened for each cellular subset. DGEs were significantly enriched in GO terms related to immune response and regulation of response to stimulus, as well as KEGG pathways such as IL-17 signaling and cytokine–cytokine receptor interaction. Intercellular communication analysis revealed enhanced communication between cell subpopulations following infection. This study demonstrated the important role of goat PBMCs during *P. multocida* infection early stage, identified key DGEs and immune pathways, and provided critical insights into the immune response mechanisms of goats to *P. multocida* infection.

## Supplementary Information

**Additional file 1: Primers and probe used for PCR and RPA-LFD.**

**Additional file 2: PCR reaction system and procedure of six kinds of bacteria.**

**Additional file 3: Establishment and optimization of an RPA-LFD assay for the detection of toxigenic** ***P. multocida***. **A** PCR amplification of the *toxA-N* gene from *P. multocida* HN01 strain. M: D2000 DNA Marker; 1: *toxA-N* gene. **B** Adenine tailing reaction for *toxA-N* gene product. M: D2000 DNA Marker; 1: *toxA-N* gene. **C** Identification of pMD19T-toxA-N positive plasmid. 1: colony PCR; 2: *toxA-N* gene PCR product; M: 1 kb DNA Ladder. **D** and **E** Optimization of reaction time (**D**) and temperature (**E**) for RPA-LFD. NC: negative control; M: D2000 DNA Marker. **F** and **G** Evaluation of sensitivity (**F**) and specificity (**G**) of RPA-LFD. NC: negative control; M: D2000 DNA Marker; P: positive control; 1: serotype A *P. multocida*; 2: serotype D *P. multocida*; 3: *Pseudomonas aeruginosa*; 4: *Brucella*; 5: *Haemophilus parahaemolyticus*; 6: *Acinetobacter baumannii*; 7: *Streptococcus*; 8: *Salmonella typhimurium*; 9: *Klebsiella acidogenes*.

**Additional file 4:** **Primers used for qRT-PCR.**

**Additional file 5:** **qRT-PCR reaction system and procedure.**

**Additional file 6:** **Bacterial detection in five goats prior to** ***P. multocida*** **challenge**. **A**-**F** Nasal swab PCR detection of *Brucella*, *P. multocida*, *Staphylococcus aureus*, *Acinetobacter baumannii*, *Klebsiella pneumoniae*, and *Mannheimia haemolytica*. **G** RPA-LFD detection of *P. multocida* in peripheral blood. **H** Detection of *Brucella* by RBPT. 1-5: tested goats; M: D2000 DNA marker; P: positive control; NC: negative control.

**Additional file 7: ELISA results of four pathogens in five goats prior to** ***P. multocida*** **infection**.

**Additional file 8: Routine blood examination results of five goats prior to** ***P. multocida*** **infection**.

**Additional file 9: Revival and identification of** ***P. multocida*** **for infectious inoculum preparation**.** A** Gram staining verification of resuscitated bacteria. **B** PCR identification of conserved gene and capsular typing genes in resuscitated bacteria. **C** and **D** 24-hour growth curve (**C**) and linear equation (**D**) of resuscitated *P. multocida*. N: negative control; M: D2000 DNA Marker; 1: *hyaD-hyaC *gene (serotype A); 2: *kmt* gene (conserved gene); 3: *dcbF* gene (serotype D).

**Additional file 10: HE staining of heart (A), liver (B), spleen (C), kidney (D) tissues from two goats following** ***P. multocida*** **infection**.

**Additional file 11: HE staining of left lung tissues from two goats following** ***P. multocida*** **infection**.** A** and **C** The HE staining of non-lesioned left lung tissues from goat No. 3 (**A**) and goat No. 5 (**C**). **B** and **D** The HE staining of lesioned left lung tissues from goat No. 3 (**C**) and goat No. 5 (**D**).

**Additional file 12: Potential marker genes in each cell types.**

**Additional file 13: Differentially expressed genes shared by the two comparison groups (Q-value＜0.05, |Log**_**2**_**(FoldChange)|＞1).**

**Additional file 14: GO enrichment analysis of 48 genes in two comparison groups (*P*-value＜0.05)**.

**Additional file 15: KEGG enrichment analysis of 48 genes in two comparison groups (*P*-value＜0.05)**.

**Additional file 16: GO enrichment analysis of differentially expressed genes between groups (Q-value＜0.05).**

**Additional file 17: KEGG enrichment analysis of differentially expressed genes between groups (Q-value＜0.05).**

**Additional file 18: Functional enrichment analysis of B cell and T cell subsets. A** and **B** GO enrichment analysis of B cell subsets in the two comparative groups. **C** and **D** KEGG enrichment analysis of B cell subsets in the two comparative groups.

**Additional file 19: Differential gene set score analysis of 21 selected KEGG pathways. **Gene set score differences for each pathway across eight major cell subpopulations (**A**) and between CK and Pm groups (**B**). The x-axis represents gene set scores, the y-axis corresponds to cell subpopulations (**A**) or groups (**B**), and *P*-values for pairwise comparisons are annotated on the right.

**Additional file 20: B and T cells pseudotime trajectory analysis of differentially expressed genes in each differentiation lineage (*P*-value＜0.01, |Log**_**2**_**(Fold Change)|＞1).**

**Additional file 21: Cell communication of significant ligand receptor pairs in CK and Pm Groups, respectively (*P*-value＜0.05)**.

**Additional file 22: Differential ligand–receptor pairs between CK and Pm group (*P*-value＜0.05)**.

**Additional file 23: Intercellular communication analysis of B cell, T cell and monocyte subpopulations in goat PBMCs pre- and post-** ***P. multocida*** **infection**.** A**-**C** Dot plots depict ligand–receptor pair counts and communication probability values between top 5 ligand cells and top 5 receptor cells within B cell subpopulations (**A**), T cell subpopulations (**B**), and integrated monocyte and subpopulations of B cells and T cells (**C**) for both CK and Pm groups. **D**-**F** The networks visualization present ligand–receptor pair counts and communication probability information across B cell subpopulations (**D**), T cell subpopulations (**E**), and integrated monocytes and subpopulations of B cells and T cells (**F**). The size of the peripheral circle is proportional to the ratio of the number of intercellular ligand–receptor pairs. The thickness of the line indicates the strength of the change in communication. In the right network diagram, the blue line indicates that the communication of the CK group is stronger, and the red line indicates that the communication of the Pm group is stronger. **G**-**I** Bubble charts displaying the top 15 cell populations ligand–receptor pairs with highest communication probability for each of the 15 most statistically significant signaling pathways based on minimal *P*-values of B cell subpopulations (**G**), T cell subpopulations (**H**), and integrated monocytes and subpopulations of B cells and T cells (**I**) in CK and Pm groups, respectively. **J** and **K** Heat maps displaying the communication patterns of B cell subpopulations (**J**) and T cell subpopulations (**K**) in PBMCs of uninfected or infected goats with *P. multocida*. The columns on the upper and right sides are the accumulation of longitudinal and transverse strength.

**Additional file 24: Directed acyclic graph (A) and KEGG metabolic pathway map (A) of DEGs from *P. multocida*-infected goat blood RNA-seq**.

**Additional file 25: Differentially expressed genes for each data type** (***P*-value＜0.05, |Log**_**2**_**(FoldChange)|＞1).**

**Additional file 26: Images of agarose gel for bacterial detection and RPA-LFD detection. A**-**F** Nasal swab PCR detection of *Brucella*, *P. multocida*, *Staphylococcus aureus*, *Acinetobacter baumannii*, *Klebsiella pneumoniae*, and *Mannheimia haemolytica*. **G** PCR amplification of the *toxA-N* gene from *P. multocida* HN01 strain. **H** Adenine tailing reaction for *toxA-N* gene product. **I** Identification of pMD19T-toxA-N positive plasmid. **J** and **K** Optimization of reaction time (**J**) and temperature (**K**) for RPA-LFD. **L** and **M** Evaluation of sensitivity (**L**) and specificity (**M**) of RPA-LFD. **N** and **O** PCR identification of conserved gene and capsular typing genes in resuscitated bacteria.

## Data Availability

All scRNA-seq data and RNA-seq data generated in this study are available in the Genome Sequence Archive in BIG Data Center, Beijing Institute of Genomics (BIG, http://gsa.big.ac.cn), Chinese Academy of Sciences (project accession no.PRJCA030346 and PRJCA031801). All the analyzed datasets in the current study are available from the corresponding author on reasonable request.
