# Supplementary material for: Profiling cell dynamic changes of goat peripheral blood mononuclear cells after Pasteurella multocida infection with single-cell transcriptomics and histopathology
Source: Vet Res. 2026 May 5;57:61. doi: 10.1186/s13567-025-01661-2 (PMC13154703; doi:10.1186/s13567-025-01661-2)
Supplement: Supplementary file 1 — Additional file 1: Primers and probe used for PCR and RPA-LFD. [file 13567_2025_1661_MOESM1_ESM.pdf]

Additional file 1. Primers and probe used for PCR and RPA-LFD.

Amplified object	Gene Name	Sequence (5' - 3')	Size	Purpose
P. multocida	*KMT1*	F: ATCCGCTATTTACCCAGTGG	460 bp	Bacterial
		R: GCTGTAAACGAACTCGCCAC		detection
Brucella	*IS711*	F: CTGGTGGGTATCGCATTACTCGG	591 bp	Bacterial
		R: TTCAGGAAAGCCTGGCGGTACTG		detection
Staphylococcus	*nuc*	F: GCGATTGATGGTGATACGGTT	278 bp	Bacterial
aureus		R: AGCCAAGCCTTGACGAACTAAAGC		detection
Acinetobacter	*parC*	F: TCGCGGAAAGCTCATCTTGT	410 bp	Bacterial
baumannii		R: TGATGCGGAAGCAGTGATGA		detection
Klebsiella	*KHE*	F: ATGAAACGACCTGATTGCATTCGC	489 bp	Bacterial
pneumoniae		R: TTACTTTTTCCGCGGCTTACCGTC		detection
Mannheimia	*lktD*	F: GCAGGAGGTGATTATTAAAGTGG	206 bp	Bacterial
haemolytica		R: CAGCAGTTATTGTCATACCTGAAC		detection
P. multocida	*dcbF*	F: TTACAAAAGAAAGACTAGGAGCCC	657 bp	Serotype
(serotype D)		R: CATCTACCCACTCAACCATATCAG		identification
P. multocida	*hyaD-hyaC*	F: TGCCAAAATCGCAGTCAG	1044 bp	Serotype
(serotype A)		R: TTGCCATCATTGTCAGTG		identification
P. multocida	*toxA-N*	F: ATGAAAACAAAACATTTTTTTAACTCAGA	1515 bp	Plasmid
		R: AGGAGGATCATCAGGTGAGATAGA		construction
P. multocida	*toxA-N*	F: GTTATCTGATACATTTCTTGCAATGGCTATTC	251 bp	Bacterial
		R: Biotin-CATCTGTATTAAGAAATTCAACAAATGGTTGTG		detection
		Probe: FAM-GGTTTGTAACACATATTATGGCAATAAGGA		(RPA-LFD)
		-THF-GAAGTTCAAATAACT-p		

Biotin: Biotin labeling; FAM: Fluorophore; THF: Tetrahydrofuran spacer; p: Phosphate group
