# Supplementary material for: Profiling cell dynamic changes of goat peripheral blood mononuclear cells after Pasteurella multocida infection with single-cell transcriptomics and histopathology
Source: Vet Res. 2026 May 5;57:61. doi: 10.1186/s13567-025-01661-2 (PMC13154703; doi:10.1186/s13567-025-01661-2)
Supplement: Supplementary file 2 — Additional file 2: PCR reaction system and procedure of six kinds of bacteria. [file 13567_2025_1661_MOESM2_ESM.pdf]

Additional file 2. PCR reaction system and procedure of six kinds of bacteria.

Bacteria	Component	Volume (μL)	Temperature (°C)	Time	
Brucella	positive control \ nasal swab	0.5\2	94	5 min	
	F	1	94	30 s	
	R	1	62	30 s	30 cycle
	2×Taq PCR MasterMix II	5	72	30 s	
	ddH$_2$O	1.5\1	72	10 min	
			16	∞	
P. multocida	positive control \ nasal swab	0.5\2	95	5 min	
(KMT1)	F	1	95	30 s	
	R	1	58	45 s	30 cycle
	2×Taq PCR MasterMix II	5	72	1 min	
	ddH$_2$O	1.5\1	72	5 min	
			16	∞	
Staphylococcus	nasal swab	2	94	4 min	
aureus	F	1	94	1 min	
	R	1	55	30 s	30 cycle
	2×Taq PCR MasterMix II	5	72	1 min 30 s	
	ddH$_2$O	1	72	4 min	
			16	∞	
Acinetobacter	positive control \ nasal swab	0.5\2	95	3 min	
baumannii	F	1	94	30 s	
	R	1	56	50 s	30 cycle
	2×Taq PCR MasterMix II	5	72	1 min 30 s	
	ddH$_2$O	1.5\1	72	5 min	
			16	∞	
Klebsiella	positive control \ nasal swab	0.5\2	95	3 min	
pneumoniae	F	1	94	1 min	
	R	1	55	45 s	30 cycle
	2×Taq PCR MasterMix II	5	72	1 min	
	ddH$_2$O	1.5\1	72	10 min	
			16	∞	

Bacteria	Component	Volume (μL)	Temperature (°C)	Time	
Mannheimia	nasal swab	2	94	5 min	
haemolytica	F	1	94	30 s	30 cycle
	R	1	60	30 s	
	2×Taq PCR MasterMix II	5	72	30 s	
	ddH_2O	1	72	5 min	
			16	∞	
