# Supplementary material for: Profiling cell dynamic changes of goat peripheral blood mononuclear cells after Pasteurella multocida infection with single-cell transcriptomics and histopathology
Source: Vet Res. 2026 May 5;57:61. doi: 10.1186/s13567-025-01661-2 (PMC13154703; doi:10.1186/s13567-025-01661-2)
Supplement: Supplementary file 3 — Additional file 3: Establishment and optimization of an RPA-LFD assay for the detection of toxigenic P. multocida. A PCR amplification of the toxA-N gene from P. multocida HN01 strain. M: D2000 DNA Marker; 1: toxA-N gene. B Adenine tailing reaction for toxA-N gene product. M: D2000 DNA Marker; 1: toxA-N gene. C Identification of pMD19T-toxA-N positive plasmid. 1: colony PCR; 2: toxA-N gene PCR product; M: 1 kb DNA Ladder. D and E Optimization of reaction time (D) and temperature (E) for RPA-LFD. NC: negative control; M: D2000 DNA Marker. F and G Evaluation of sensitivity (F) and specificity (G) of RPA-LFD. NC: negative control; M: D2000 DNA Marker; P: positive control; 1: serotype A P. multocida; 2: serotype D P. multocida; 3: Pseudomonas aeruginosa; 4: Brucella; 5: Haemophilus parahaemolyticus; 6: Acinetobacter baumannii; 7: Streptococcus; 8: Salmonella typhimurium; 9: Klebsiella acidogenes. [file 13567_2025_1661_MOESM3_ESM.pdf]

Establishment and optimization of an RPA-LFD assay for the detection of toxigenic *P. multocida*

The pMD19T-toxA-N plasmid was constructed as a positive control. The Recombinase-aid Amplification (RAA)-nfo nucleic acid amplification kit (Zhongce, China) was used for recombinase polymerase amplification - lateral flow dipstick (RPA-LFD) detection of *P. multocida*. The reaction system consisted of 1 tube of reaction dry powder, 38.9 μL A Buffer, 2 μL upstream primer, 2 μL downstream primer, 0.6 μL probe, 4 μL template, and 2.5 μL B buffer. The templates were the DNA of the sample to be tested, the positive plasmid, and the nuclease-free waters (as negative control). After optimizing the reaction time and temperature, and conducting specificity and sensitivity tests, the optimized reaction conditions were finally used for subsequent detection of *P. multocida*. Primers and probe sequences were detailed in Additional file 1. The results demonstrated that the established assay successfully detected toxigenic *P. multocida*, with optimal reaction conditions determined to be 30 min of incubation at 35°C. The limit of detection was found to be 10^{-1} copies/μL.

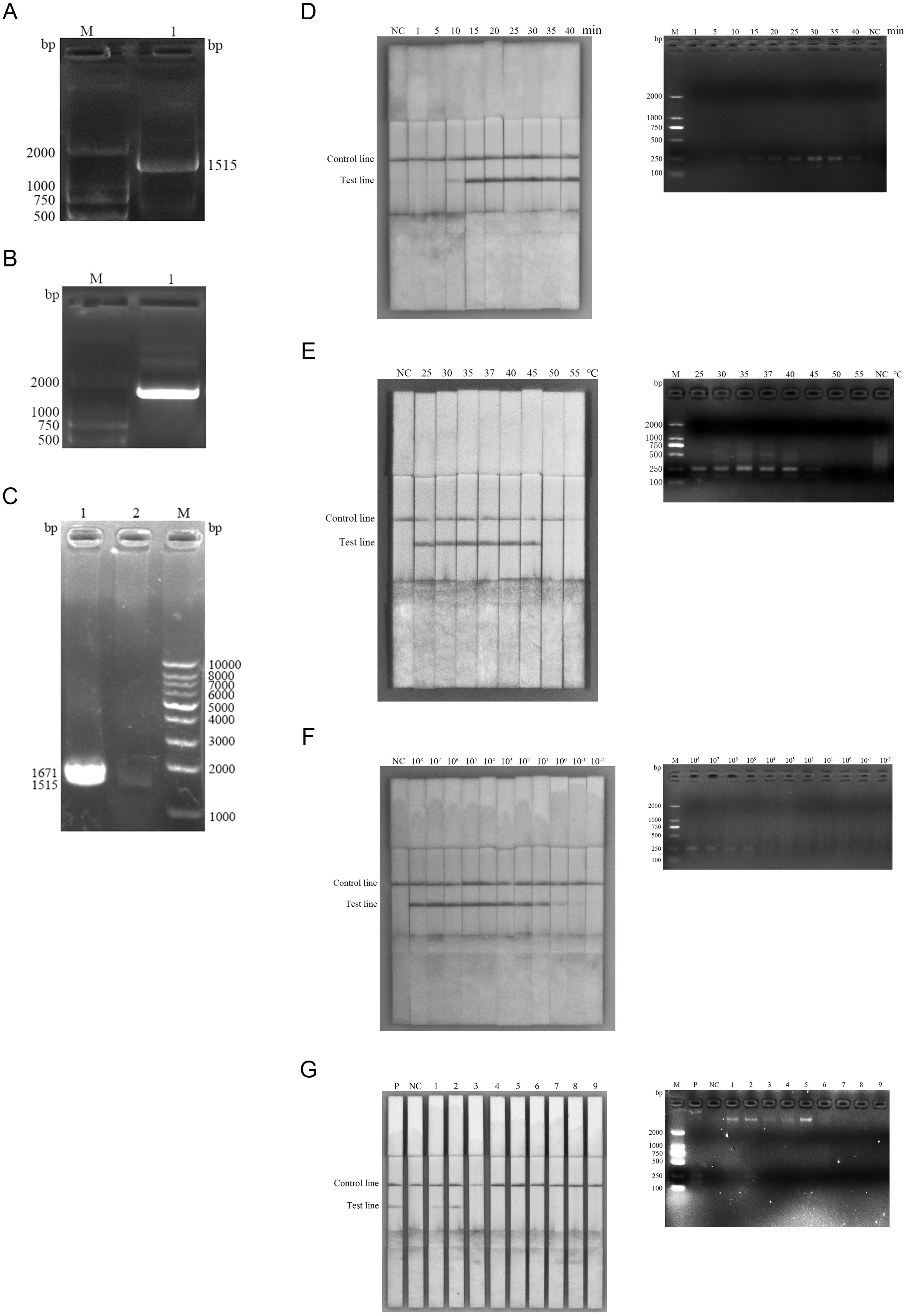

A
M
1
bp
2000
1000
750
500
1515
B
M
1
bp
2000
1000
750
500
C
1
2
M
bp
10000
8000
7000
6000
5000
4000
3000
2000
1000
1671
1515
D
NC 1 5 10 15 20 25 30 35 40 min
Control line
Test line
M 1 5 10 15 20 25 30 35 40 NC min
bp
2000
1000
750
500
250
100
E
NC 25 30 35 37 40 45 50 55 °C
Control line
Test line
M 25 30 35 37 40 45 50 55 NC °C
bp
2000
1000
750
500
250
100
F
NC 10^8 10^7 10^6 10^5 10^4 10^3 10^2 10^1 10^0 10^-1 10^-2
Control line
Test line
M 10^8 10^7 10^6 10^5 10^4 10^3 10^2 10^1 10^0 10^-1 10^-2
bp
2000
1000
750
500
250
100
G
P NC 1 2 3 4 5 6 7 8 9
Control line
Test line
M P NC 1 2 3 4 5 6 7 8 9
bp
2000
1000
750
500
250
100
