# Supplementary material for: Profiling cell dynamic changes of goat peripheral blood mononuclear cells after Pasteurella multocida infection with single-cell transcriptomics and histopathology
Source: Vet Res. 2026 May 5;57:61. doi: 10.1186/s13567-025-01661-2 (PMC13154703; doi:10.1186/s13567-025-01661-2)
Supplement: Supplementary file 4 — Additional file 4: Primers used for qRT-PCR. [file 13567_2025_1661_MOESM4_ESM.pdf]

**Additional file 4. Primers used for qRT-PCR.**

| Gene Name | Sequence (5' - 3') | Size |
|---|---|---|
| *IL1R2* | F: ACTATACCTGTGCCATGCCG | 244 bp |
| | R: TAGGCATCCTCTATGCGGGT | |
| *ZBTB21* | F: TTCTTCGTCCCTGTTCGTGG | 162 bp |
| | R: TTCTCCTCGTCCTCGGTGAT | |
| *NR1H3* | F: CTGAAGACGTCCGCGATTGAG | 283 bp |
| | R: TGTGTTGCAGCCTCTCTACC | |
| *AXL* | F: CAACCTCCATCCTCACACCC | 275 bp |
| | R: CCTCTCGCTTTAGCCCTACG | |
| *GAPDH* | F: CCTGGAGAAACCTGCCAAGTA | 141 bp |
| | R: AAGGTAGAAGAGTGAGTGTCGC | |
