# Supplementary material for: Profiling cell dynamic changes of goat peripheral blood mononuclear cells after Pasteurella multocida infection with single-cell transcriptomics and histopathology
Source: Vet Res. 2026 May 5;57:61. doi: 10.1186/s13567-025-01661-2 (PMC13154703; doi:10.1186/s13567-025-01661-2)
Supplement: Supplementary file 5 — Additional file 5: qRT-PCR reaction system and procedure. [file 13567_2025_1661_MOESM5_ESM.pdf]

Additional file 5. qRT-PCR reaction system and procedure.

Purpose	Component	Volume (μL)	Temperature (°C)	Time	
reverse	5×FastKing-RT SuperMix	4	45	15 min	
transcription	RNA	5	95	3 min	
	RNase-Free ddH_2O	11			
qPCR	cDNA	2	95	30 s	
	F	0.4	95	5 s	40 cycle
	R	0.4	60	30 s	
	2×SYBR Green Pro Taq HS Premix	10			
	RNase-Free ddH_2O	7.2			
