# Supplementary figures and images for: Profiling cell dynamic changes of goat peripheral blood mononuclear cells after Pasteurella multocida infection with single-cell transcriptomics and histopathology
Source: Vet Res. 2026 May 5;57:61. doi: 10.1186/s13567-025-01661-2 (PMC13154703; doi:10.1186/s13567-025-01661-2)

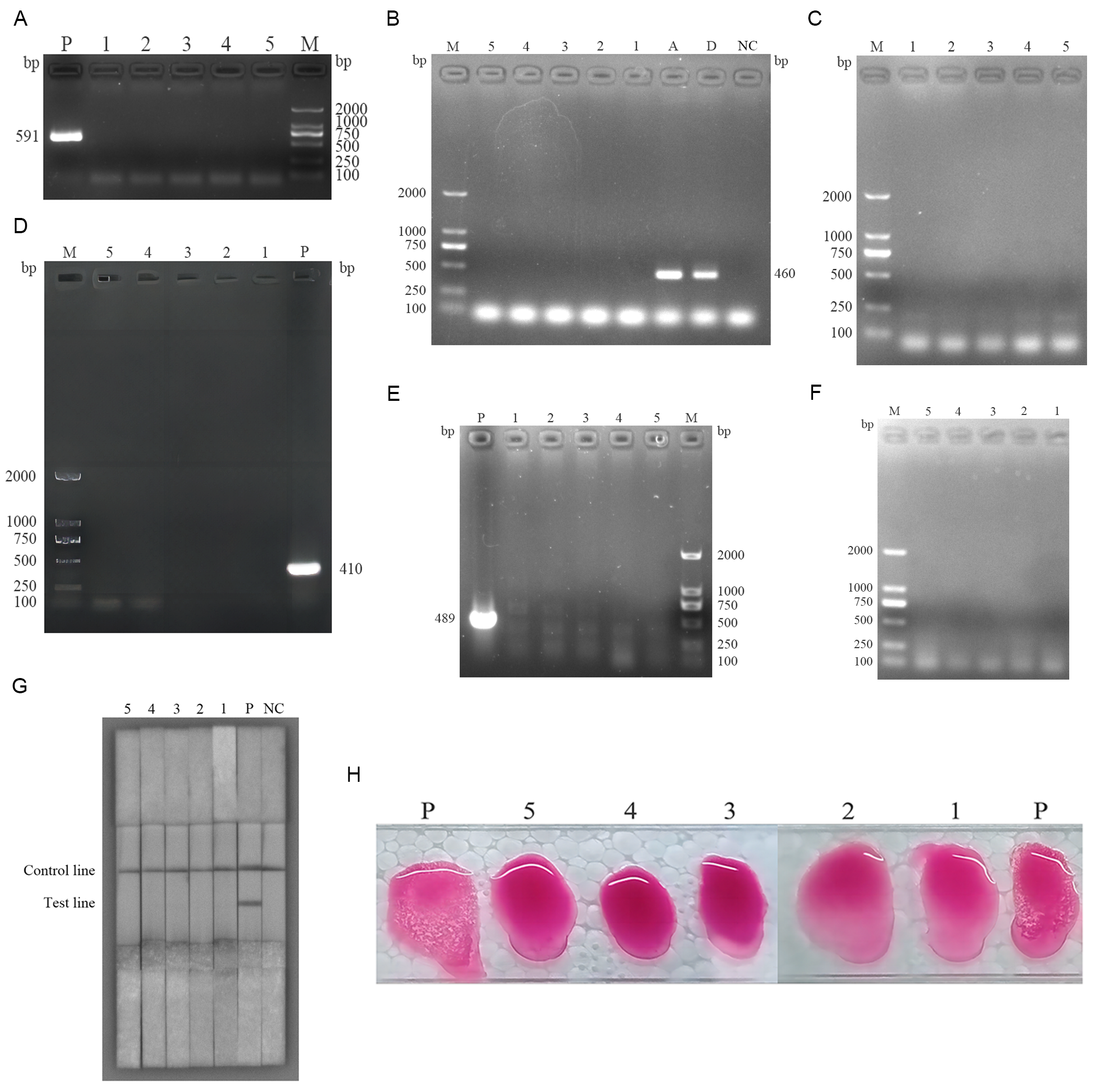

Supplement: Supplementary file 6 — Additional file 6: Bacterial detection in five goats prior to P. multocida challenge. A-F Nasal swab PCR detection of Brucella, P. multocida, Staphylococcus aureus, Acinetobacter baumannii, Klebsiella pneumoniae, and Mannheimia haemolytica. G RPA-LFD detection of P. multocida in peripheral blood. H Detection of Brucella by RBPT. 1-5: tested goats; M: D2000 DNA marker; P: positive control; NC: negative control. [file 13567_2025_1661_MOESM6_ESM.tiff]

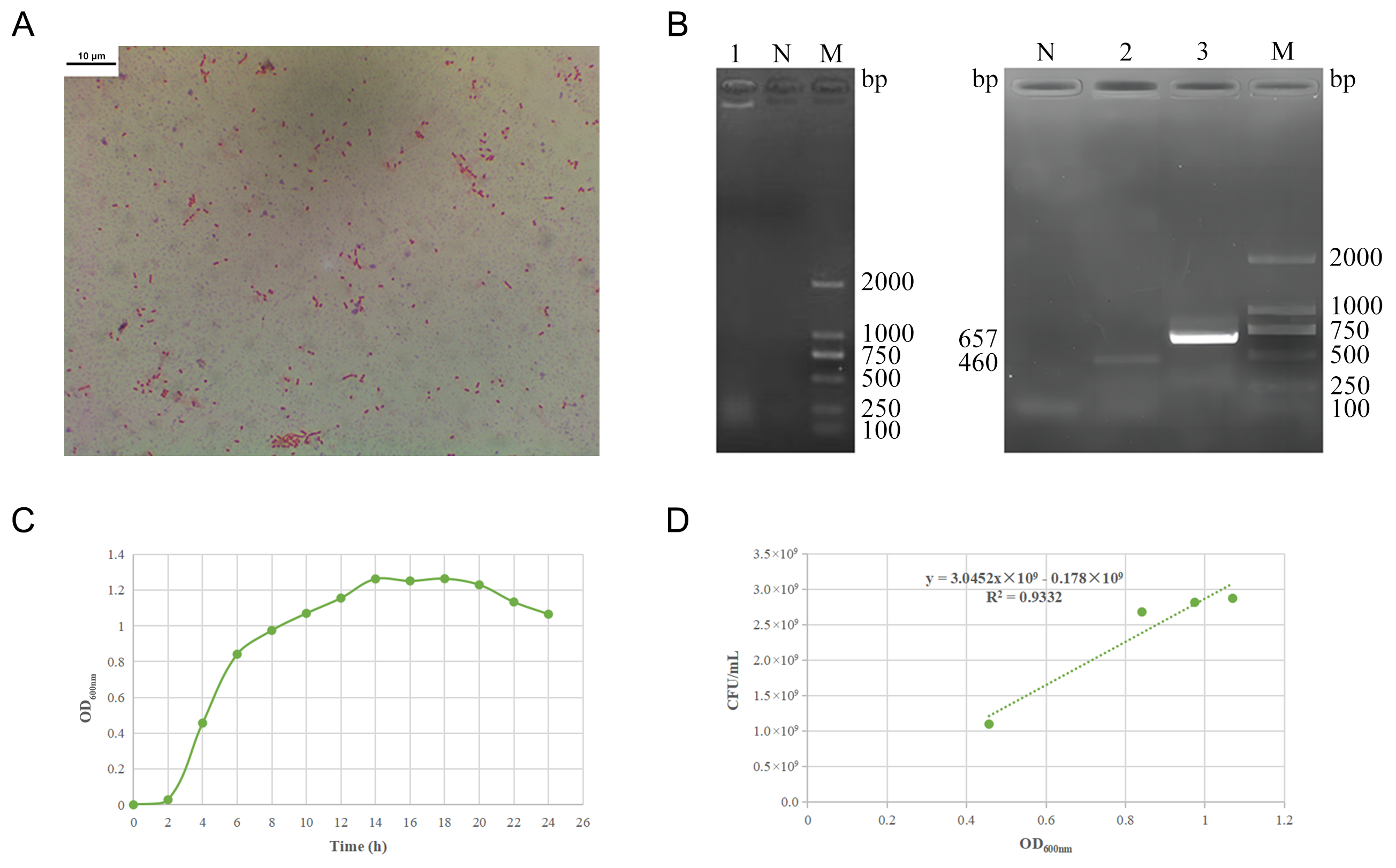

Supplement: Supplementary file 9 — Additional file 9: Revival and identification of P. multocida for infectious inoculum preparation. A Gram staining verification of resuscitated bacteria. B PCR identification of conserved gene and capsular typing genes in resuscitated bacteria. C and D 24-hour growth curve (C) and linear equation (D) of resuscitated P. multocida. N: negative control; M: D2000 DNA Marker; 1: hyaD-hyaC gene (serotype A); 2: kmt gene (conserved gene); 3: dcbF gene (serotype D). [file 13567_2025_1661_MOESM9_ESM.tif]

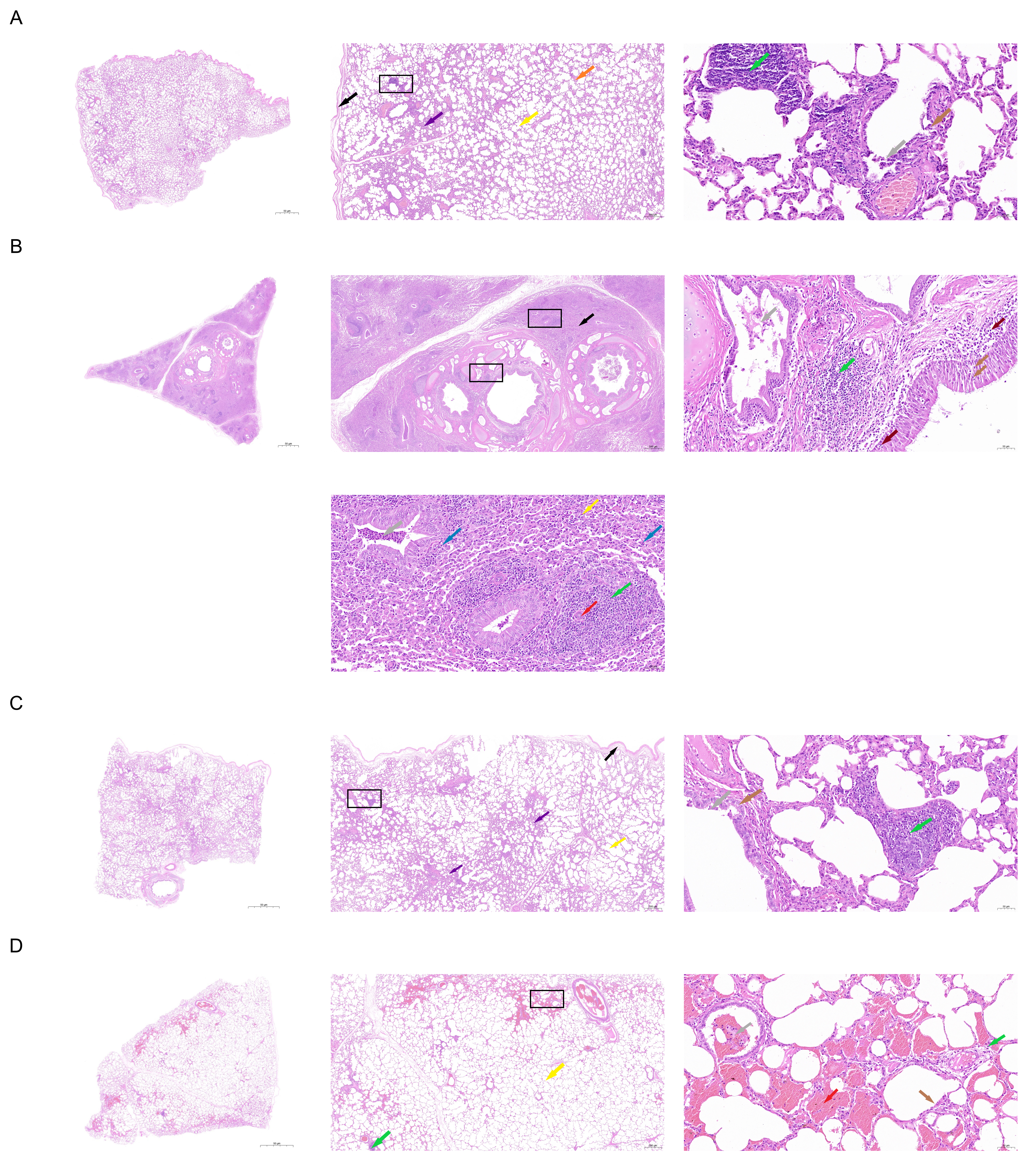

Supplement: Supplementary file 11 — Additional file 11: HE staining of left lung tissues from two goats following P. multocida infection. A and C The HE staining of non-lesioned left lung tissues from goat No. 3 (A) and goat No. 5 (C). B and D The HE staining of lesioned left lung tissues from goat No. 3 (C) and goat No. 5 (D). [file 13567_2025_1661_MOESM11_ESM.tiff]

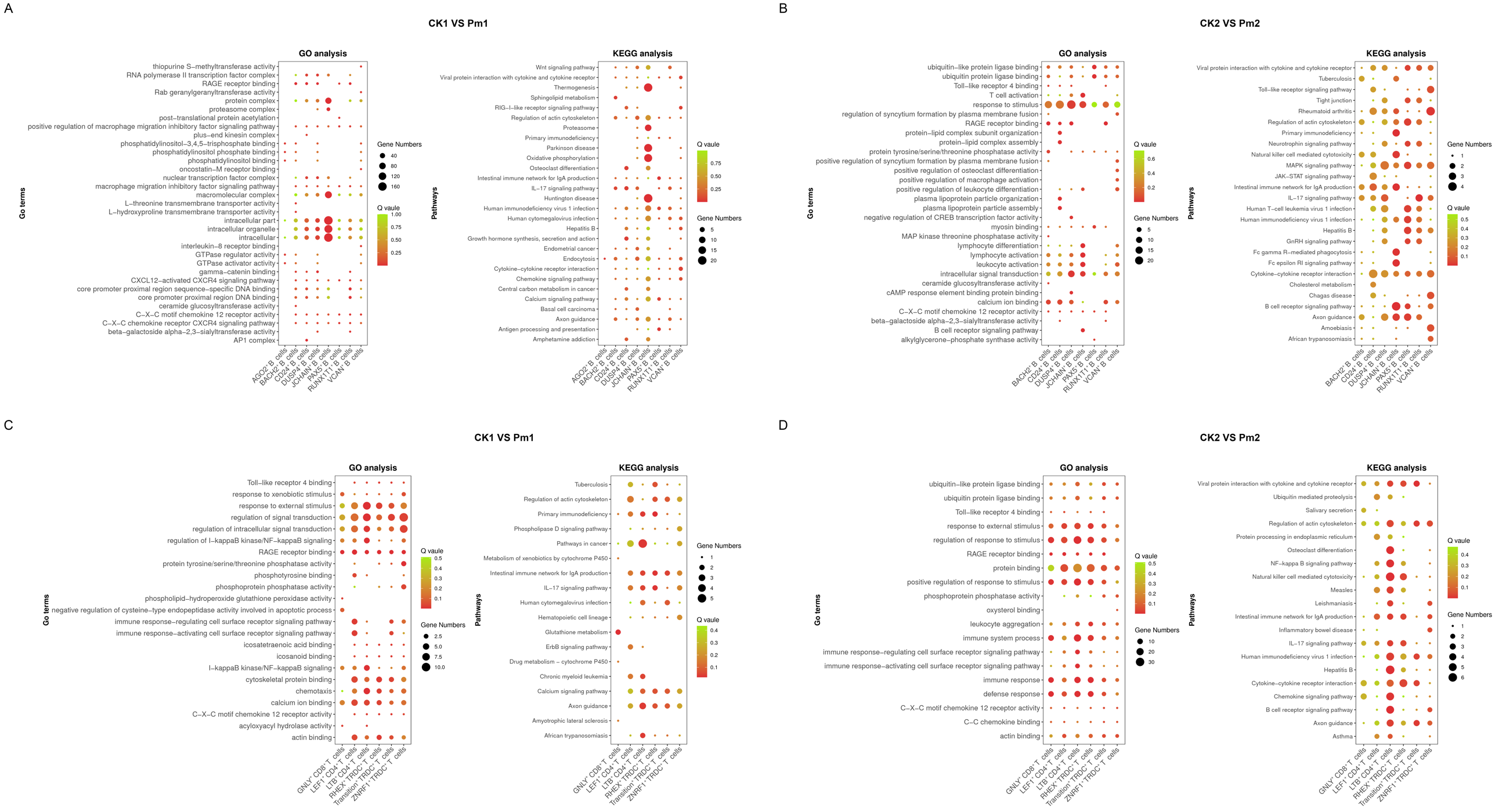

Supplement: Supplementary file 18 — Additional file 18: Functional enrichment analysis of B cell and T cell subsets. A and B GO enrichment analysis of B cell subsets in the two comparative groups. C and D KEGG enrichment analysis of B cell subsets in the two comparative groups. [file 13567_2025_1661_MOESM18_ESM.tiff]

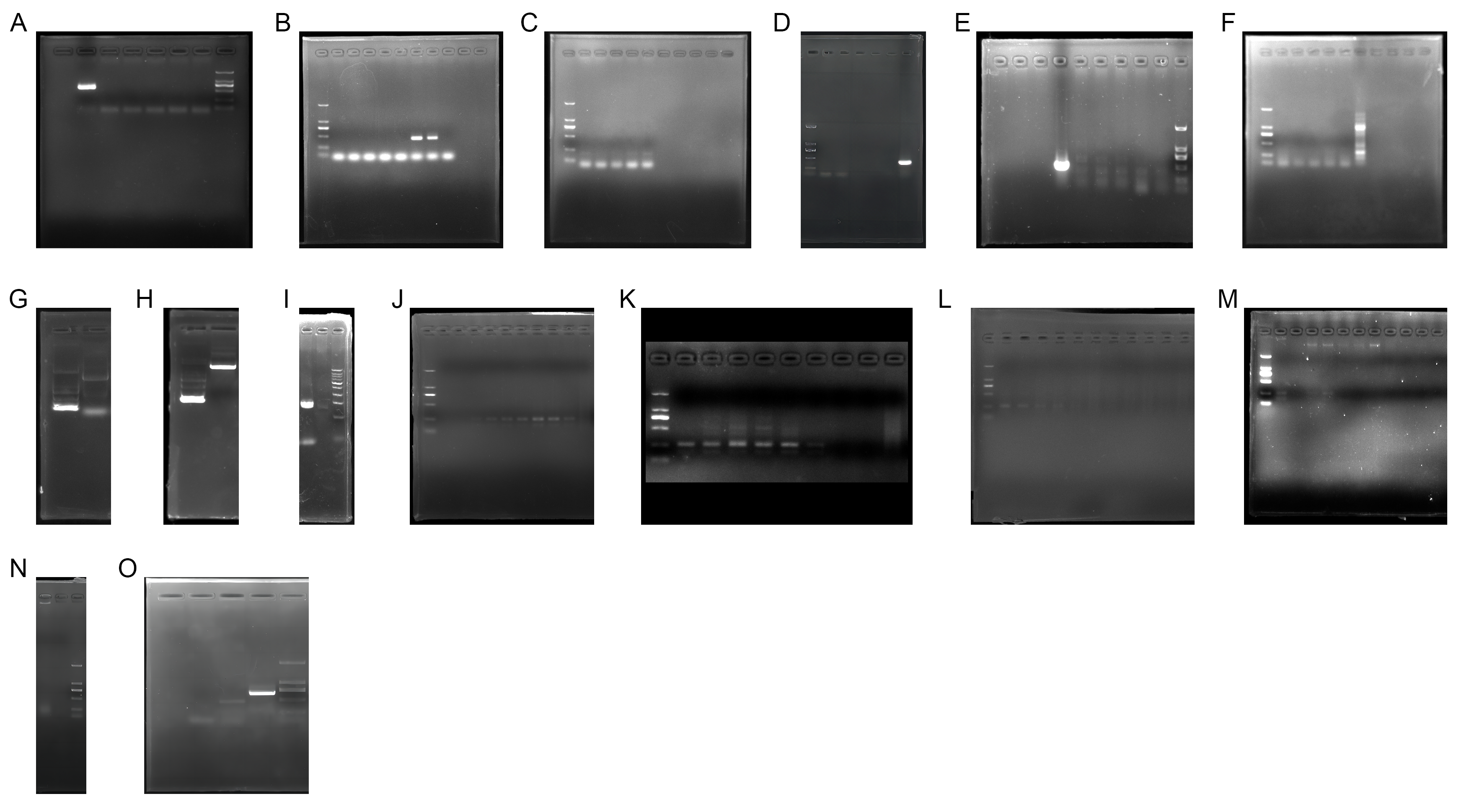

Supplement: Supplementary file 26 — Additional file 26: Images of agarose gel for bacterial detection and RPA-LFD detection. A-F Nasal swab PCR detection of Brucella, P. multocida, Staphylococcus aureus, Acinetobacter baumannii, Klebsiella pneumoniae, and Mannheimia haemolytica. G PCR amplification of the toxA-N gene from P. multocida HN01 strain. H Adenine tailing reaction for toxA-N gene product. I Identification of pMD19T-toxA-N positive plasmid. J and K Optimization of reaction time (J) and temperature (K) for RPA-LFD. L and M Evaluation of sensitivity (L) and specificity (M) of RPA-LFD. N and O PCR identification of conserved gene and capsular typing genes in resuscitated bacteria. [file 13567_2025_1661_MOESM26_ESM.tiff]
