# Supplementary material for: Profiling cell dynamic changes of goat peripheral blood mononuclear cells after Pasteurella multocida infection with single-cell transcriptomics and histopathology
Source: Vet Res. 2026 May 5;57:61. doi: 10.1186/s13567-025-01661-2 (PMC13154703; doi:10.1186/s13567-025-01661-2)
Supplement: Supplementary file 7 — Additional file 7: ELISA results of four pathogens in five goats prior to P. multocida infection. [file 13567_2025_1661_MOESM7_ESM.pdf]

Additional file 7. ELISA results of four pathogens in five goats prior to *P. multocida* infection.

Goat Number	Mean Absorbance			
	Brucella	*P. multocida*	*Mycoplasma*	*Chlamydia*
First Detection				
1	0.067	0.066	0.069	0.061
2	0.063	0.064	0.066	0.056
3	0.088	0.063	0.090	0.068
4	0.056	0.063	0.080	0.060
5	0.059	0.064	0.068	0.057
Negative Control	0.054	0.058	0.061	0.054
Blank Control	0.056	0.067	0.066	0.056
Positive Control	2.177	2.320	2.297	2.324
Second Detection				
1	0.057	0.056	0.058	0.053
2	0.057	0.057	0.057	0.052
3	0.056	0.105	0.088	0.072
4	0.062	0.073	0.085	0.064
5	0.057	0.139	0.067	0.052
Negative Control	0.058	0.054	0.056	0.050
Blank Control	0.056	0.053	0.058	0.051
Positive Control	1.903	2.243	2.188	2.292
