# Supplementary material for: Profiling cell dynamic changes of goat peripheral blood mononuclear cells after Pasteurella multocida infection with single-cell transcriptomics and histopathology
Source: Vet Res. 2026 May 5;57:61. doi: 10.1186/s13567-025-01661-2 (PMC13154703; doi:10.1186/s13567-025-01661-2)
Supplement: Supplementary file 8 — Additional file 8: Routine blood examination results of five goats prior to P. multocida infection. [file 13567_2025_1661_MOESM8_ESM.pdf]

Additional file 8. Routine blood examination results of five goats prior to *P. multocida* infection.

Detecting Item	Goat Number					Unit	Lower limit of reference value	Upper limit of reference value
	1	2	3	4	5			
Number of leukocytes (WBC)	23.83	12.53	16.27	12.1	13.08	10^9/L	5.8	25
Percentage of neutrophils (Neu%)	59.2	52.8	53.3	43.5	34	%	13	58
Percentage of lymphocytes (Lym%)	32.2	37.9	36.8	50.6	62	%	35	83
Percentage of monocytes (Mon%)	7	3.9	6.6	4.7	2.4	%	0	11
Percentage of eosinophils (Eos%)	1.5	5.4	3.3	1.1	1.5	%	0	8
Percentage of basophils (Bas%)	0.1	0.0	0.0	0.1	0.1	%	0	2.5
Number of neutrophils (Neu)	14.11	6.62	8.68	5.27	4.45	10^9/L	2.12	10.1
Number of lymphocytes (Lym)	7.68	4.75	5.99	6.13	8.11	10^9/L	3.12	22.1
Number of monocytes (Mon)	1.67	0.48	1.07	0.56	0.32	10^9/L	0	1.42
Number of eosinophils (Eos)	0.35	0.68	0.53	0.13	0.19	10^9/L	0	1.32
Number of basophils (Bas)	0.02	0.00	0.00	0.01	0.01	10^9/L	0	0.35
